# A TGFβ-ECM-Integrin signalling axis drives structural reconfiguration of the bile duct to promote polycystic liver disease

**DOI:** 10.1126/scitranslmed.abq5930

**Published:** 2023-09-13

**Authors:** Scott H Waddell, Yuelin Yao, Paula Olaizola, Alexander Walker, Edward J Jarman, Konstantinos Gournopanos, Andreea Gradinaru, Ersi Christodoulou, Philippe Gautier, Melissa M Boerrigter, Massimiliano Cadamuro, Luca Fabris, Joost PH Drenth, Timothy J Kendall, Jesus M Banales, Ava Khamseh, Pleasantine Mill, Luke Boulter

**Affiliations:** 1 MRC Human Genetics Unit, Institute of Genetics and Cancer, University of Edinburgh- Edinburgh- UK, EH4 2XU; 2 School of Informatics- University of Edinburgh- Edinburgh- UK, EH8 9AB; 3 Department of Liver and Gastrointestinal Diseases, Biodonostia Health Research Institute – Donostia University Hospital, University of the Basque Country (UPV/EHU), San Sebastian, Spain, 20014; 4 Department of Gastroenterology and Hepatology, Radboud University, Nijmegen Medical Center- 6525 GA Nijmegen- Netherlands; 5 Department of Medicine, University of Padua, 35128 Padua, Italy; 6 Department of Molecular Medicine, University of Padua, 35128 Padua, Italy; 7 Digestive Disease Section, Yale University School of Medicine, New Haven, CT 06510, USA; 8 Centre for Inflammation Research, Queens Medical Research Institute, University of Edinburgh, Edinburgh, UK EH16 4TJ; 9 National Institute for the Study of Liver and Gastrointestinal Diseases, CIBERehd, “Instituto de Salud Carlos III”, 28029 Madrid, Spain; 10 Department of Biochemistry and Genetics, School of Sciences, University of Navarra, 31008 Pamplona, Spain; 11 IKERBASQUE, Basque Foundation for Science, 48009 Bilbao, Spain; 12 Cancer Research UK Scotland Centre, Institute of Genetics and Cancer, Edinburgh, UK, EH4 2XU

## Abstract

The formation of multiple cysts in the liver occurs in a number of isolated monogenic diseases or multisystemic syndromes, during which bile ducts develop into fluid-filled biliary cysts. For patients with polycystic liver disease (PCLD), non-surgical treatments are limited and managing life-long abdominal swelling, pain, and increasing risk of cyst rupture and infection is common. We demonstrate here that loss of the primary cilium on postnatal biliary epithelial cells (via the deletion of the cilia gene *Wdr35*) drives ongoing, pathological remodelling of the biliary tree, resulting in progressive cyst formation and growth. The development of cystic tissue requires the activation of transforming growth factor-β (TGFβ) signalling, which promotes the expression of a pro-cystic, fibronectin-rich extracellular matrix and which itself is perceived by a changing profile of Integrin receptors on the cystic epithelium. This signalling axis is conserved in liver cysts from patients with either autosomal dominant polycystic kidney disease or autosomal dominant polycystic liver disease, indicating there are common cellular mechanisms for liver cyst growth regardless of the underlying genetic cause. Cyst number and size can be reduced by inhibiting TGFβ or Integrin signalling *in vivo* and we suggest that our findings represent a therapeutic route for patients with polycystic liver disease, the majority of which would not be amenable to surgery.

## Introduction

**Figure 1:**
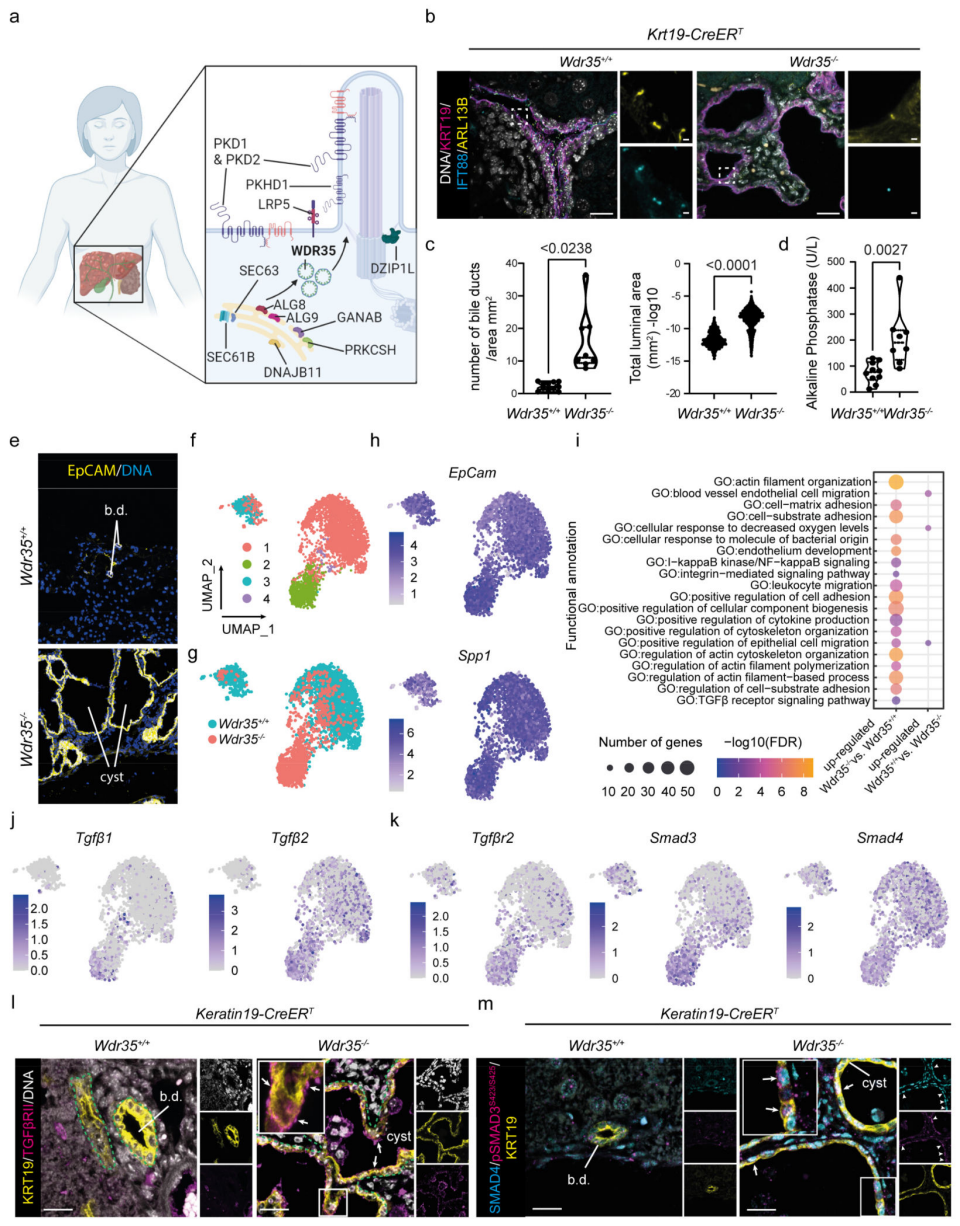
Selective loss of *Wdr35* in BECs results in PC loss and the formation of polycystic liver disease. **A.** The mutations in genes associated with PKD *(PKD1, PKD2, PKHD1, ALGG9, DNAJB11, DZIP1L, GANAB, LRP5);* ADPLD *(PRKCSH, SEC63, SEC61B, GANAB, ALG8*, and *LRP5)*, and syndromic cystic diseases (for example *WDR35)* in relation to the primary cilium. **B.** Cilia (or cilia-rudiments after *Wdr35-*deletion) are marked by ARL13B (yellow) and IFT88 (cyan) in KRT19-positive cells (magenta), DNA in grey. **C.** Number *(Wdr35^+/+^* n=11 animals and *Wdr35^-/^* n=8 animals) and area of ducts or cysts in 12-month *Wdr35^+/+^* (n=812 ducts) and *Wdr35^-/-^* (n=3834 cysts). **D.** Serum alkaline phosphatase (ALP) concentrations of 12-month *Wdr35^+/+^* (n=10) and *Wdr35^-/-^* (n=8) mice. **E.** EpCAM-positive BECs in *Wdr35^+/+^* and *Wdr35^-/-^* livers (yellow). **F.** scRNA-seq UMAP showing Seurat clusters 1-4 into which *Wdr35^+/+^* and Wdr35^-/-^ BECs segregate. **G.** UMAP showing segregation of *Wdr35^+/+^* (cyan) and *Wdr35^-^* (salmon) cells**. H.** *EpCam* and *Spp1* mRNA abundance across all cells **I.** GO term analysis of cluster 1 (87% composed of *Wdr35^+/+^* cells) and cluster 2 (97% composed of *Wdr35^-/-^* cells). **J to K.** UMAPs of *Tgfβ1, Tgfβ2* (J) and *Tgfβr2, Smad3*, and *Smad4* (K) in *Wdr35^+/+^* (n=3060) and *Wdr35^-/-^* (n=966) cells. **L to M.** Immunohistochemistry of *Wdr35^+/+^* and *Wdr35^-/-^* livers stained for KRT19 (yellow) TGFBRII (magenta, L) or pSMAD3^S423/S425^ (magenta, M), and SMAD4 (cyan). DNA is grey. White arrows denote positive BEC staining. Green dotted lines denotes duct or cyst boundary (scale bar=100 μm).

Pathological liver cysts are an important comorbidity in multiple diseases and syndromes(1, 2) driven by dysfunction of the primary cilium (PC), a complex sensory organelle that protrudes from the apical surface of biliary epithelial cells (BECs)(3, 4). The essential nature of PC in liver development(5, 6) makes understanding the molecular role of this organelle in the structural maintenance of the adult bile duct challenging. Genetic alterations in PC genes result in hepatorenal fibrocystic diseases such as hereditary autosomal dominant and autosomal recessive polycystic kidney diseases, which are primarily caused by mutations in *PKD1, PKD2* and *PKHD1* (7–9). In autosomal dominant polycystic liver disease, in which cysts form only within the liver, most causative genes encode ER-associated proteins, such as *PRKCSH* and *SEC63*, that play roles in protein transport, folding and trafficking to PC (10–13), (cyst genetics covering hepatic fibropolycystic disease in autosomal dominant polycystic kidney or liver disease and autosomal recessive polycystic kidney disease collectively referred to as PCLDs), summarised in Figure 1a and reviewed in (14). Ultimately, evidence from human disease genetics strongly implicates PC dysfunction as the root cause of hepatic cyst development.

Complex fibropolycystic features are also associated with syndromic ciliopathic disease in which key PC genes, such as intraflagellar transport (IFT) genes *(WDR35, WDR19*, and *IFT56)* that are required to build and maintain functional PC are mutated (15–17). Across these diseases, cysts form throughout the liver, however due to the complexity of these syndromes and their presentation in other organs, the presence of hepatic cysts is likely under-estimated (18). Furthermore, it remains unclear whether BEC PC-loss *per se* in the adult is sufficient to promote liver cystogenesis.

Here, we show that PC loss in adult mouse BECs is sufficient to cause bile duct expansion, driving cyst formation through *de novo* production of a laminin- and fibronectin-rich pro-cystic microenvironment. This newly formed niche promotes both cell-autonomous changes in cell shape and duct-level mechanical rearrangements that converge to drive cyst-fission, a process whereby single, large cysts undergo morphological splitting. This process gives rise to many, smaller polycystic progeny and can be halted by pharmacological inhibition of specific pro-cystic integrin receptors, thereby providing a potential approach to treat patients living with liver cysts.

## Results

### Loss of primary cilia results in tissue remodelling to form TGFβ-high cysts

Previous reports suggest that in liver cysts from autosomal dominant polycystic kidney disease, PC length decreases with cyst size and are absent in large *cysts*(19), and in rats with genetic *Pkhd1-loss* BEC cilia are shorter and dysmorphic (20). Furthermore, in the kidney, loss of PC (through the genetic deletion of *Thm1*, which encodes tetratricopeptide repeat protein 21B/TCC21B, a component of the IFT-A complex) leads to the formation of renal cysts, though deletion of the same gene in the liver does not automatically lead to hepatic cyst formation, indicating that different tissues are variously sensitive to cilia loss (21-23). To determine whether loss of functional PC was sufficient to promote the formation of cysts within the liver *per se*, we deleted the WD Repeat Domain 35 (*Wdr35*)/Intraflagellar Transport 121 *(Ift121)* gene specifically in postnatal BECs (using 6-8 week-old *Krt19-CreER^T^* mice, fig. S1A). We found that loss of *Wdr35*-alone was sufficient to induce formation of hepatic cysts (fig. S1b). WDR35/IFT121 is part of a highly conserved retrograde IFT-A complex that we have shown is essential for the transport of ciliary membrane cargo (such as ARL13B) and necessary for PC elongation and signalling (24). This approach enables us to define the roles of PC specifically in adult biliary homeostasis, separating whether changes in PC *per se* cause PCLD rather than errant ductal plate patterning observed in embryonic cilia mutants (5, 23). In control *Krt19-CreER^T^;Wdr35^+/+^* (referred to as *Wdr35^+/+^)* bile ducts, PC protrude from the apical surfaces of BECs and are positive for IFT-B complex (IFT88-positive) and the ciliary membrane-localised protein ARL13B; however in *Krt19-CreER^T^;Wdr35^flox/flox^* mice (abbreviated to *Wdr35*^-/-^) after the administration of tamoxifen, BEC PC are largely absent and instead exist as IFT88-positive, shortened rudiments lacking ARL13B and normal PC function, as we have previously described (24) (Fig. 1b and fig. S1c).

In *Wdr35^+/+^* mice, bile ducts are arranged around portal veins forming a network of small, pan- cytokeratin positive ductules. After deletion of *Wdr35* from BECs, pan-cytokeratin positive ducts became polycystic (fig. S1D and S1E), with a small increase in proliferation (fig. S1F and S1G) and onset of progressive polycystic disease between 6 and 12 months after the loss of ARL13B-positive cilia (fig. S1H, S1I and fig. S2). Of note, although both male and female patients develop liver cysts, there is a higher incidence of liver cysts in female patients (25). Accordingly, female *Wdr35^-/-^* animals had more cysts after 12 months than males, possibly reflecting the sexual dimorphism seen in patients (fig. S2A- C). The increase in both the number of cystic ducts and their area (Fig. 1C and fig. S2) is accompanied with an elevation of serum alkaline phosphatase concentrations, indicating that PC loss and the formation of liver cysts leads to biliary inflammation (Fig. 1D) without affecting other hepatic functions (fig. S3).

Although symptomatic liver cysts are a comorbid factor in multiple diseases, little is known about the molecular processes that enable pathological cysts within the liver to form and grow. We therefore sought to define the molecular profiles of cystic BECs following *Wdr35* loss. CD45-/CD31-/EpCAM+ BECs from *Wdr35^+/+^* or *Wdr35^-/-^* livers were isolated 12 months after PC loss (Fig. 1E and fig. S4A and S4B), once polycystic disease was established and during the ongoing pathological remodelling of the biliary tree. Using 10X single-cell RNA sequencing (scRNA-seq) we analysed the gene expression of 3,060 normal and 966 cystic cells. BECs clustered into 4 distinct and highly data file populations (Fig. 1F and fig. S5A-C) based on hierarchical clustering in which normal and cystic cholangiocytes clustered separately at the transcriptional level (Fig. 1G). RNA velocity (26, 27) predicted that cystic cells are derived from a subset of normal BECs as expected, rather than via transformation from other hepatic cell types (fig. S6A). Within the four clusters identified, the majority of *Wdr35^+/+^* cells fell within cluster 1, whereas most *Wdr35^-/-^* cells occupied cluster 2 (cluster 3 and 4 are made of fewer cells and are a mixture of cells from both WT and mutant animals, fig. S6B). Cells within clusters 1-4 had distinct transcriptional profiles and were enriched for diverse gene ontology (GO) terms, including regulation of cell morphogenesis and actin filament organisation (fig. S6C and S6D). Furthermore, *Wdr35^-/-^* cystic BECs expressed specific unique markers including *Pdpn* (Podoplanin) and *Vcam1* (Vascular Cell Adhesion Molecule-1), which were not expressed by *Wdr35^+/+^* cells (fig. S7A and S7B). *Wdr35^-/-^* BECs did not appear to change lineage (into hepatocytes, for example) and retained a number of BEC-specific markers including *EpCAM* (on which BECs were sorted), the pan-BEC marker *Spp1* (Fig. 1H and fig. S8A), as well as *Cftr, Clu, Sox9, Hnf1b, Krt19*, and *Tspan8* (fig. S8B). For further analysis, only BECs expressing both *EpCam* and *Spp1* were analysed. A large number of genes were differentially expressed between clusters 1 and 2 (data file S1) and gene ontology (GO) term analysis identified multiple transcriptional signatures that were enriched in *Wdr35^-/-^* cells, including Ca^2+^ and MAPK signalling, which have been associated with the formation of both renal (7) and hepatic (28) cysts (fig. S9A-C). Hedgehog signalling, which is tightly controlled by PC in other contexts (22, 29), was not altered in cystic cells (fig. S9D). Rather, we identified biological processes previously found to be important in biliary development, repair, and cystogenesis; GO terms associated with cytoskeleton remodelling and cell-matrix adhesion (30, 31), namely transforming growth factor-β (TGFβ)(32) and integrin (33) signalling were changed (Fig. 1I and data file S2), adding further support to the idea that alterations in the extracellular matrix (ECM)(34) and cytoskeletal dynamics, in addition to induction of known cystogenic signalling signatures, promote the generation of cysts *in vivo*(35, 36).

Canonical TGFβ promotes SMAD3 phosphorylation and with SMAD4 regulates inflammatory cytokines and ECM gene transcription. In the liver, TGFβ-SMAD signalling is a central driver of biliary fibrosis(37), but is constrained to nascent portal fibroblasts surrounding ducts that produce collagen in response to TGFβ(38). In contrast, our data suggests that cystic BECs themselves may be targets of TGFβ ligands. We sought therefore to define the role of TGFβ in liver cyst formation. In addition to upregulation of *Tgfb1* and *Tgfb2* ligands (Fig. 1J), cystic BECs upregulated transcription of the receptor *Tgfbr2* and the downstream effector of this pathway *Smad3*, whereas the obligate binding partner of SMAD3, *Smad4*, was widely expressed across both cystic and non-cystic BECs (Fig. 1K and fig. S10A). Using immunofluorescence, we confirmed TGFBRII expression and localisation: *Wdr35^+/+^* BECs did not express TGFBRII, however 12 months after *Wdr35* loss, TGFBRII was apically enriched on cystic BECs and also present on large, intraluminal immune cells (Fig. 1L and fig. S10B). To test whether the presence of TGFBRII on cystic BECs was indicative of cysts being TGFβ responsive, we examined SMAD expression. In control *Wdr35^+/+^* bile ducts, SMAD4 was present but lowly expressed and, with the exception of a small numbers of cells, (active) pSMAD3^S423/S425^ was absent. In contrast, in cystic BECs, high nuclear SMAD4 and extensive pSMAD3^S423/S425^ staining was observed (Fig. 1M). Active SMAD signalling in the epithelium of autosomal dominant polycystic kidney disease renal cysts has been described (39), suggesting there could be common TGFβ-mechanisms of cyst growth between organs. To test this, we assessed cystic liver tissue from patients with PCLD where either the causative mutation had not been identified or were driven by mutations in *SEC63* or *PRKCSH* (both encoding ER proteins involved in the biogenesis and trafficking of polycystin proteins(12)) (fig. S11A). In these genetically diverse human pathologies, elevated nuclear SMAD4 and pSMAD3^S423/S425^ were detected in cystic BECs, confirming that TGFβ signalling is a common hallmark of liver cyst formation (fig. S11B).

### *A* pro-cystic extracellular matrix drives cyst formation

**Figure 2:**
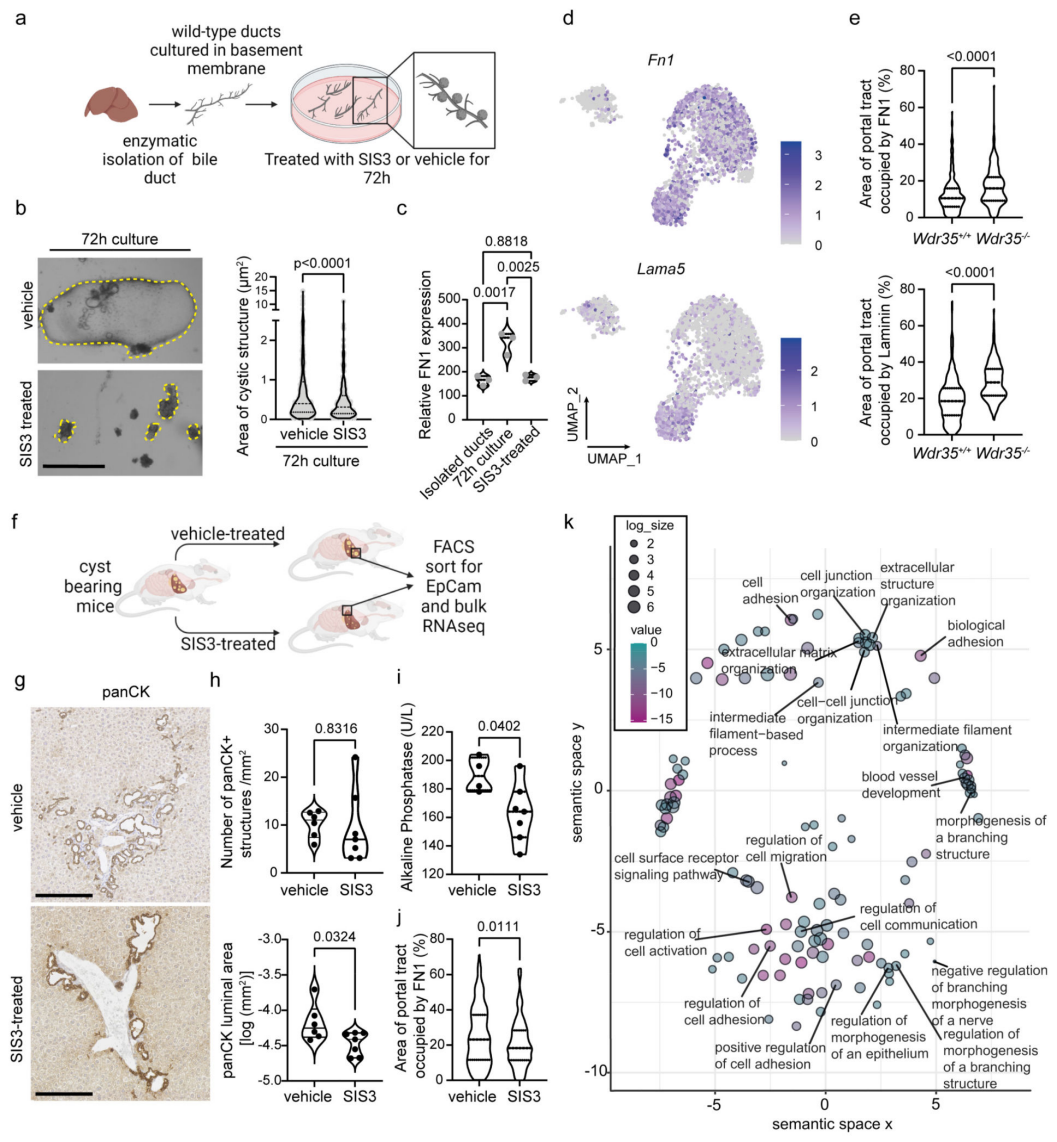
Cystic epithelial cells are sensitised to TGFβ signalling thereby promoting the formation of a pro-cystic ECM. **A.** Schematic of duct-to-cyst cultures **B.** Photomicrographs showing typical structures of ducts after 72 h culture with and without SIS3 treatment. Quantification of *ex vivo* cultures treated with the SMAD3-inhibitor SIS3 (vehicle: n=487 and SIS3: n=378). Yellow dotted line denotes the duct boundary (scale bar=50 μm). **C.** Protein expression of fibronectin assessed by RPPA in freshly isolated ducts or after 72h culture with vehicle or SIS3-treatment (n=3 biological replicates). **D.** UMAP showing *Fn1* and *Lamb3* expression. **E.** Quantification of immunohistological expression of fibronectin (284 *Wdr35^+/+^* ducts from n=11 animals and 237 *Wdr35^-/-^* ducts from n=8 animals) and pan-laminin (177 *Wdr35^-/-^* cysts from n=11 animals and299 *Wdr35^+/+^* cysts from n=8 animals). **F.** Schematic detailing the treatment of cyst-bearing *Wdr35^-/-^* mice with SIS3. **G.** Immunohistochemical staining for panCK-positive BECs (scale bar=500 μm)**. H.** Number and log size of panCK-positive structures in cyst-bearing mice treated with vehicle alone or SIS3 for 3 weeks (vehicle: n=6 animals and SIS3: n=7 animals). **I.** Serum alkaline phosphatase concentrations in cyst-bearing mice treated with vehicle (n=4 animals) or SIS3 (n=7 animals). **J.** Fibronectin positivity in *Wdr35^-/-^* animals treated with SIS3 (277 cysts from n= mice) or vehicle (196 cysts from n= 7 mice)**. K.** REViGO output showing the rationalised GOterms following GOrilla analysis.

As TGFβ signalling is exclusively activated in cystic BECs, we postulated that TGFβ-SMAD is necessary for cystic growth. We adapted previous liver organoid methodologies (40) to isolate bile ducts from wild type mice. These *ex vivo* ducts were cultured in Matrigel and formed sphere-like cystic structures within 72 h (Fig. 2A). In this assay, treatment of ducts with the SMAD3-inhibitor SIS3 reduced the area of cysts, and rather than developing into dilated cystic structures, ducts remained as tubes with closed ends (Fig. 2B). We confirmed this was concomitant with reduced abundance of SMAD3 (fig. S11C), implying that cyst formation is SMAD3-dependent. Indeed, when normal human BECs or BECs derived from the liver cysts of patients with autosomal dominant polycystic liver or kidney disease were treated with SIS3 *in vitro*, we found that the growth of cystic epithelial cells was inhibited with lower amounts of SIS3 than their normal counterparts (fig. S12)

Given the failure of SIS3-treated ducts to form cysts and evidence from our BEC-specific scRNA-seq data, we hypothesised that signalling through SMAD3 enables BECs to alter their physical microenvironment, thereby promoting cystogenesis. To test this, we isolated total protein extracts from freshly isolated bile ducts or ducts cultured for 72 h and treated with either SIS3 or a vehicle alone. Using a custom Reverse Phase Protein Array (RPPA), a sensitive antibody-based proteomic approach biased to detect changes in ECM and cell-adhesion proteins, we found that when compared to freshly isolated ducts, 72 h cultured ducts increased their fibronectin production as they became cystic. Furthermore, the amount of fibronectin produced was inhibited in cultures that had been treated with SIS3 (Fig. 2C).

The fibronectin gene, *Fn1*, is a target of SMAD3 *in vitro* (41) and our data from both mouse and human cells (GSE145271) indicated that BECs alter their own microenvironment via TGFβ-SMAD3 by inducing fibronectin expression, in addition to a range of other classical targets of TGFβ-SMAD signalling *(SERPINE1, ZEB2, SNAI1/2, TWIST2)* as well as ECM components including collagens and laminins (fig. S11D and S11E). Fibronectin acts as a nucleating protein for fibrillar collagens (including collagen-I and collagen-IV), which interact with laminins to promote formation of the basement membrane(42). *Wdr35^-/-^* cystic BECs demonstrated increased expression of *Fn1, Lama5, Lamb3, Lamc1, Col4a1* and *Col4a2*, but not *Col1a1* or *Col1a2* when compared to *Wdr35^+/+^* BECs (Fig. 2D, fig. S13A and S13B and data file S1). In addition to these transcriptional changes, we confirmed the ECM surrounding *Wdr35^-/-^* cysts is different from *Wdr35^+/+^* bile ducts, showing an expanded basement membrane with increased fibronectin and laminins (Fig. 2E).

Given our *ex vivo* duct culture showed BEC-derived fibronectin promotes cyst expansion via SMAD3, we administered 6-month cyst-bearing *Wdr35^-/-^* animals the SMAD3 inhibitor SIS3 or vehicle alone for 3 weeks (Fig. 2F) to determine whether *in vivo* inhibition of SMAD3 signalling impacts the production of fibronectin and progression of cystogenesis. SMAD3 inhibition did not reduce the number of panCK-positive cysts in the *Wdr35^-/-^* liver; however, it reduced cyst size (Fig. 2G and 2H). Moreover, SMAD3-inhibition ameliorated pathogenic biliary alterations as evidenced by reduction of serum alkaline phosphatase (Fig. 2I) and glutamate dehydrogenase (fig. S14) and was associated with a reduction in fibronectin deposition around cysts after SIS3-treatment (Fig. 2J). Last, we took a pathway-agnostic approach to examine how TGFβ-SMAD3 regulates cyst formation in PCLD. CD45-/CD31-/EpCAM+ BECs were isolated from *Wdr35^-/-^* cysts treated with either SIS3 or vehicle and subjected to bulk RNA sequencing. Upon SIS3 treamtent, cystic BECs were transcriptionally distinct from those isolated from vehicle animals (data file S3). Analysis with GOrilla and REViGO (data file S4) showed that SIS3-treatment changes gene sets associated with tubular morphogenesis, branching and organisation of the ECM, reiterating that SMAD3 regulates a range of biological processes required for cyst generation from bile ducts through the reactivation of morphogenetic processes (Fig. 2K).

### Changes in ductular architecture are driven by cyst-specific integrins

**Figure 3:**
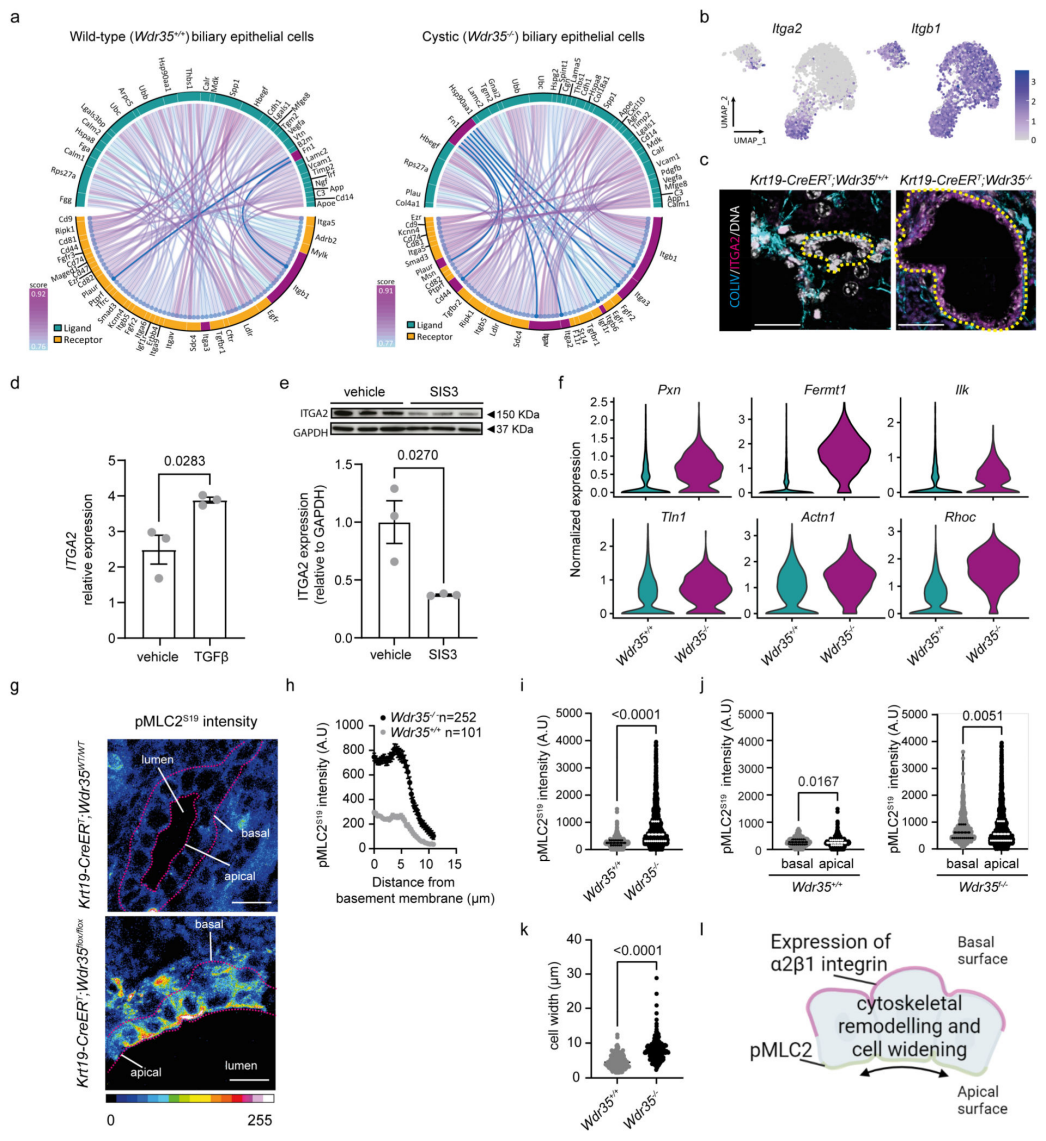
Cystic cells expand their ability to interact with a fibronectin-rich microenvironment and alter their cell shape. **A**. Ligand-receptor interactions in scRNA-seq data from *Wdr35^+/+^* and *Wdr35^-/-^* cells. **B.** UMAPs showing *Itga2* and *Itgb1* mRNA expression. **C.** Immunohistochemistry for Collagen-IV (cyan) and ITGA2 (magenta) in *Wdr35^-/-^* or *Wdr35^+/+^* BECs. Yellow dotted line denotes duct/cyst boundary. (scale bar=50 μm). **D.** mRNA abundance of *ITGA2* in a human H69 cells after stimulation with recombinant-TGFβ for 16 h (n=3). **E**. Protein abundance of ITGA2 in H69 cells treated with 10 μM of SIS3 for 72 h. Histogram shows GAPDH-normalised ITGA2 expression (n=3). **F.** scRNA-seq expression of integrin effector molecules *(Pxn, Fermt1, Ilk, Tln1, Actn1, Rhoc)* comparing median transcript abundance between *Wdr35^+/+^* and *Wdr35^-/-^* cells. **G.** Intensity projection of pMLC2 staining in *Wdr35* ducts and *Wdr35^-/-^* BECs. Dotted line denotes duct and cyst boundary. (scale bar=20 μm). **H.** Quantification of pMLC2 intensity across the apico-basal axis of normal biliary cells (grey) and cystic epithelial cells (black). **I.** Average pMLC2 intensity in *Wdr35^+/+^* and *Wdr35^-/-^* cells. **J.** Apical and basal pMLC2 abundance in *Wdr35^+/+^* and *Wdr35^-/-^* biliary cells (for **H-J**, n=101 *Wdr35^+/+^* cells and n=252 *Wdr35^-/-^* cells, n=4 animals per group). **K.** Width of biliary cells from *Wdr35^+/+^* (165 cells from n=4 animals) and *Wdr35^-/-^* (172 cells from n= 4 animals) mice.

How cystic BECs receive and interpret signals from this changing extracellular niche remains unclear. In heterocellular scRNA-seq data, receptor-ligand interaction modelling predicts functional interactions between different cell-types (43, 44). Here, using SingleCellSignalR (43) we sought to define whether there are cell-type autonomous signals between cystic BECs. Within wild-type BECs, we identified a number of receptor-ligand interactions (Fig. 3A and data file S5), notably between *Fn1*, encoding for fibronectin, and *Itgb1* (β1-integrin), a canonical ECM receptor which heterodimerises with other α-integrin subunits to sense alterations in the ECM. Analysis of cystic *Wdr35^-/-^* BECs also identified candidate interactions between *Itgb1* and *Fn1*, however this *Fn1* domain expanded to include putative *Wdr35^-/-^*-specific interactions with *Itga2, Itga3, Itgav, Itgb6, Cd44*, and *Plaur* (Fig. 3A and data file S6). As integrin-receptor affinity is altered depending on the relative expression of both α- and β- subunits (45), we clustered wild-type and cystic BECs based on genotype (*Wdr35^+/+^* vs *Wdr35^-/-^)* to define which integrin subunits were most differentially expressed between these groups (fig. S15A). β1-integrin *(Itgb1)* transcripts were universally expressed by BECs whether they were normal or cystic, whereas α2-integrin *(Itga2)* mRNA was selectively expressed by *Wdr35^-/-^* mutant cells (Fig. 3B and figure S15B). Integrin-α2β1 is a relatively promiscuous integrin receptor that binds laminins, fibrillar collagens, and fibronectin (46). In addition to specific transcriptional upregulation of *Itga2*, both ITGB1 and ITGA2 proteins were expressed on cystic BECs from *Wdr35^-/-^* mice, whereas Wdr35^+/+^ BECs only expressed ITGB1 (Fig. 3C, fig. S15B). As TGFβ-SMAD signalling promotes cyst formation by changing the production of ECM components-we postulated that TGFβ-signalling may also alter the ability of BECs to sense these changes. Indeed, BECs treated with recombinant TGFβ upregulated integrin mRNA expression, including *ITGA2* (Fig. 3D and fig. S11D). Moreover, SIS3-mediated inhibition of SMAD3 in BECs reduced ITGA2 protein abundance *in vitro* and *in vivo* (Fig. 3E and fig. S15c and S15d). In addition to cell-surface integrin receptors, downstream mediators of integrin signalling *Pxn, Fermt1, Ilk, Tln1, Actn,1* and *Rhoc* were also upregulated in *Wdr35^-/-^* cystic BECs (Fig. 3F). In other ductular systems, ECM integrin signalling regulates the actin cytoskeleton, thereby modulating cellular width and apico-basal tension (47–49). In liver cysts, BECs lost their cuboidal shape and adopted a flattened morphology, indicative of changes in intracellular mechanics (fig. S1I). Additionally, BECs showed a generalised increase phosphorylated myosin light chain 2 (pMLC2) expression compared to their wild-type counterparts (Fig. 3G-I). In *Wdr35^+/+^* BECs, low amounts of pMLC2 were preferentially localised to the basal domain of the cell, however cystic BECs had increased apical pMLC2 abundance (Fig. 3J) which was also associated with an increase in BEC width (from lateral membrane to lateral membrane, Fig. 3K). Together, our data support that loss of PC signalling in BECs leads to profound cell-autonomous morphogenetic changes, driving cyst formation in the adult liver. It is likely that cysts grow through active sensing of a pro-cystic ECM and cytoskeletal remodelling, thereby driving cyst growth.

As in our mouse model of PCLD, patients with cystic liver diseases have high amounts of ITGA2 (fig. S16A) and pMLC2 (fig. S16B) on cystic BECs, suggesting ciliary or trafficking-mediated disruptions converge on similar pathomechanisms (50). Critically, these patients carry pathogenic mutations covering a range of autosomal dominant polycystic liver *(SEC63* and *PRKCSH)* and kidney *(PKD1* and *PKD2)* disease-related genes, suggesting that the acquisition of integrin-α2β1 mediated-sensing of the microenvironment may be a common mechanism during cyst formation across pathologies regardless of the underlying genetics.

### Polycystic disease forms from the morphogenic division of parental cysts

Patients with PCLD, by definition, present with multiple (>10) cysts in their liver; however, it remains unclear whether each cyst originates clonally from mutant precursor cells (corresponding to many separate originating events) or whether single cysts themselves grow and then structurally divide to produce a polycystic pattern of disease (in which cells of origin are divided between multiple cysts). Indeed, in *Wdr35^-/-^* livers, where some cysts appeared largely spherical in shape, other cysts readily formed septa that protruded into or across the cyst lumen at both 6 and 12 m (fig. S17A). These results indicate active tissue remodelling in established cysts and that single large cysts can divide to give rise to daughter cysts. Indeed, using wholemount imaging we found multiple examples of polycystic regions forming through structural narrowing and division of pre-existent cysts (fig. S17B).

**Figure 4:**
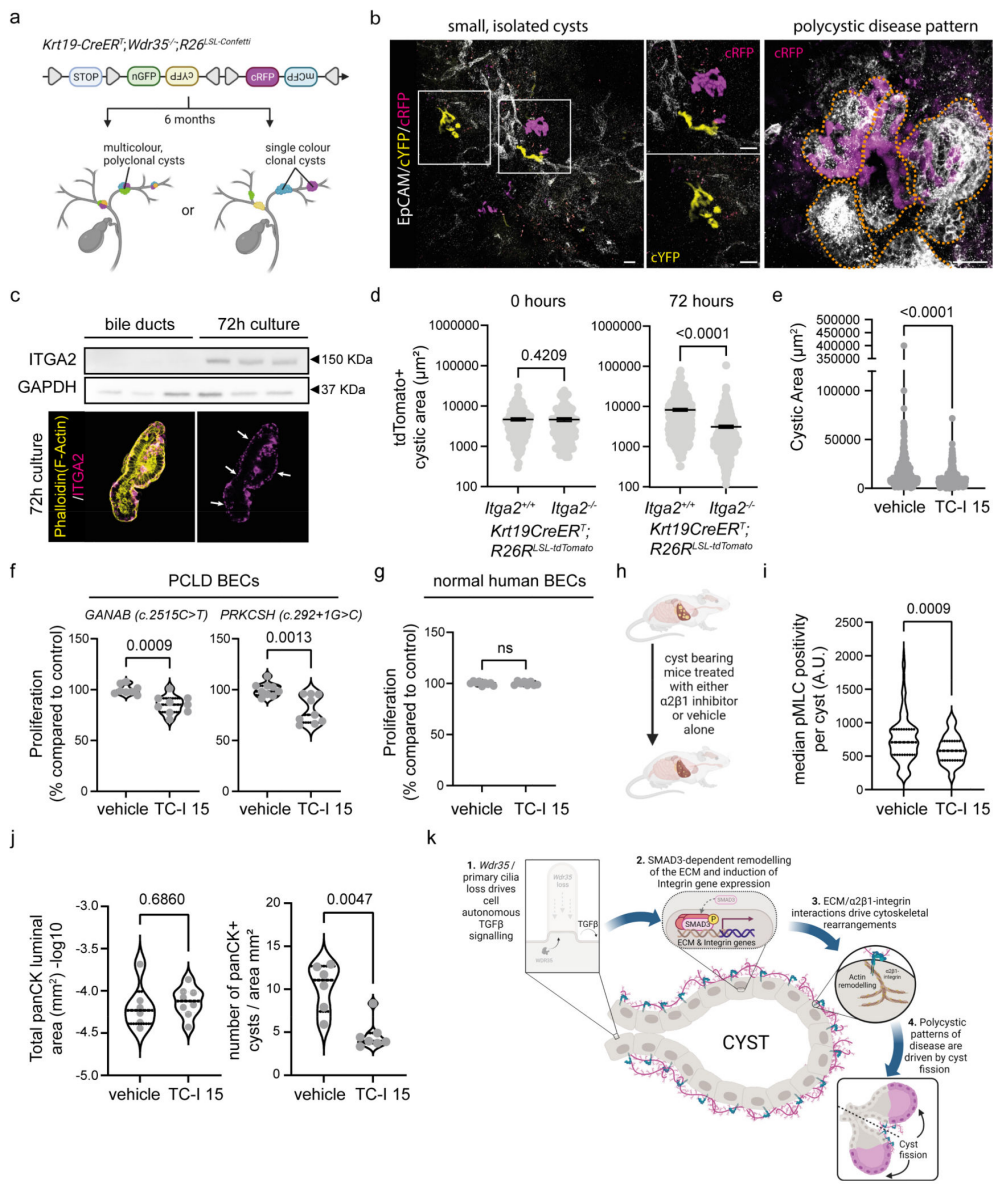
Integrin-α2β1 promotes hepatic cystogenesis by mediating cyst fission. **A.** Schematic representation of the potential lineage traced outcomes of murine, *Wdr35-/-* cysts in the *Krt19- CreER^T^;Wdr35^-/-^;R26^LSL-Confetti^* transgenic line. **B.** Wholemount imaging of EpCAM (grey), cYFP (yellow), or cRFP (magenta). White boxes represent magnified regions of interest. Cysts sharing a single mutant clone denoted by dotted orange line. (Scale bar=100 μm) **C.** ITGA2 and GAPDH immunoblots from freshly isolated bile duct extracts versus those cultured for 72 h. Immunocytochemistry of ITGA2 (magenta) and F-actin (phalloidin; yellow) in 72 h cultured ducts (white arrows show basally-localised ITGA2, scale bar=50μm). **D.** Area of *Itga2^+/+^* (N=138) versus *Itga2^-/-^* (N=114) tdTomato-positive cultured ducts upon plating or following 72 h culture, *(Itga2^+/+^:* N=304 versus *Itga2^-/-^:* N=569). **E.** Area of cultured ducts when treated with 100 μM TC-I 15 (N=439) or vehicle (N=354) for 72 h. **F.** Proliferation of BECs from patients with PCLD treated with TC-I 15 or vehicle (N=9 experimental replicates). **G.** Healthy human BECs treated the same way as in F, (N=9 experimental replicates). **H.** Schematic of the *in vivo* treatment of cyst-bearing mice with TC-I 15 (21 days at 20 mg/kg). **I.** Median expression of pMLC2^S19^ staining in cystic cells treated with vehicle (176 cysts from n=7 mice) or TC-I 15 (73 cysts from n=6 mice). **J.** Quantification of cystic luminal area or number of cysts in the livers of *Wdr35^-/-^* mice treated with TC-I 15 (n=6 mice) or vehicle (n=7-8 mice).

Previous work has demonstrated that ductal plate malformation during embryogenesis or loss-of- heterozygosity in causative polycystic genes is required for cyst formation (51–53). In other epithelial systems, loss-of-heterozygosity (in tumour suppressor genes, for example) drives clonal selection (54, 55). To determine whether the cysts that form following *Wdr35*-loss are derived from multiple individual clonal events or because of the physical division of cysts, we crossed *K19CreER^T^; Wdr35^flox/flox^* mice with the Confetti lineage reporter mouse line *(K19CreER^T^; Wdr35^flox/flox^; R26^LSL-Confetti^;* abbreviated to *Wdr35^-/-^*-Confetti), (fig. S18A). In this model, upon CRE recombination cells are stochastically labelled with one of nuclear GFP (nGFP), membranous CFP (mCFP), cytoplasmic YFP or RFP (cYFP/cRFP), or no fluorescence (56), thereby enabling clonal tracing of recombined BECs within cysts (Fig. 4A). Six months after *Wdr35*-deletion (and Confetti labelling), livers were surveyed using 3D confocal imaging using the FUnGI protocol(57). In *Wdr35^-/-^*-Confetti mice, small, isolated cysts were never completely clonal in colour and were comprised of both labelled and unlabelled cells (Fig. 4B and fig. S18B). Where clusters of cysts had formed (corresponding to regions of polycystic disease), adjacent cyst walls often shared the same label colour (Fig. 4B and fig. S18B). These results strongly imply that stereotypical polycystic disease pattern forms from multiple mutant cells and larger cysts may undergo cyst-fission and “pinch off” to generate smaller, daughter cysts To explore this further, we adapted our *ex vivo* duct culture and found that as ducts became cysts, BECs expressed ITGA2 protein *de novo*, which localised to the basal (non-luminal) surface of cysts, whereas non-cultured ducts were ITGA2-negative (Fig. 4C and fig. S20C).

To directly test whether the expression of ITGA2 functionally promotes the transition from normal ducts into cysts, we isolated bile ducts from *Krt19CreER^T^* mice also carrying a *R26^LSL-tdTomato^* reporter allele (to define which cells had activated Cre) and *Itga2^flox/flox^* (or *Itga2^+/+^* as a control). Cre was activated *in vivo* by administering mice tamoxifen and 7 days after the final dose, ducts were isolated and cultured in Matrigel (from hereon in defined at *Itga2^-/-^)*. Immediately after plating (0 h), tdTomato-positive ducts from *Itga2^-/-^* mice had the same dimensions as those from *Itga2^+/+^* animals. After 72 h in culture, the tdTomato-positive cysts forming from *Itga2^-/-^* ducts were smaller than in *Itga2^+/+^* controls (Fig. 4D), suggesting that ITGA2 is required for cysts to grow. To validate this, we treated wild-type *ex vivo* cultures with the integrin-α2β1 inhibitor TC-I 15, which also reduced the area of cystic structures that formed compared to vehicle-treated cultures alone (Fig. 4E).

As patients with liver cysts had a consistent upregulation of ITGA2 in cystic epithelial cells (fig. S16A) and cyst formation from cultured ducts is limited when integrin-α2β1 is inhibited, we postulated that pharmacological inhibition of integrin-α2β1 could selectively reduce the expansion of cystic cells and leave wild-type cells intact. Indeed, normal human cholangiocytes or cystic cholangiocytes isolated from patients with autosomal dominant polycystic kidney *(GANAB* (c. 2515 C>T) mutation) or liver *(PRKCSH* (c. 292+1G>C)) disease and treated with TC-I 15 for 48 h demonstrated a reduction in ITGA2 protein (fig. S20A). However, only cystic cholangiocytes demonstrated reduced proliferation (Fig. 4F), which was not the case for TC-I 15-treated normal human BECs (Fig. 4G and fig. S19A). Notably, treatment with TC-I 15 did not induce apoptosis in either wild-type or PCLD BECs (fig. S19B).

To test whether integrin-α2β1 inhibition *in vivo* was sufficient to halt or reverse disease progression, 6-month-old cyst-bearing *Wdr35^-/-^* mice were dosed with TC-I 15 or vehicle alone for 3 weeks (Fig. 4H). In treated animals, the amount of ITGA2 protein expressed by cystic BECs in vivo was reduced (fig. S20B) and pMLC2 staining in cystic BECs was decreased compared to untreated cysts, indicating that integrin-α2β1 signalling *in vivo* directly underlies the cytoskeletal remodelling observed in cystic epithelial cells (Fig. 4I). Treatment of cyst-bearing animals with TC-I 15 did not reduce cyst size in these animals (in fact, cysts were moderately larger, suggestion disruption to normal cystic tissue dynamics), however fewer cysts formed after treatment without an appreciable difference in serum biochemistry (Fig. 4J and fig. S20D). Together, this indicates that in polycystic disease, cysts undergo active, integrin-α2β1-dependent fission and this targetable cell autonomous process drives disease progression (fig S21).

### Discussion

Hepatorenal fibrocystic diseases include the most common monogenic diseases in the world (4, 58). The genes that, when mutated, give rise to these conditions are well defined but heterogeneous and converge on the form and function of primary cilia. Despite this, our understanding of the cellular processes that drive cystogenesis particularly in the liver is lacking and as such, we have struggled to define unifying mechanisms by which hepatic cystogenesis can arise. Using a mouse model of hepatic cystogenesis driven by the postnatal deletion of the cilia gene *Wdr35* specifically in bile ducts, we generated a scRNA-seq dataset of PCLD. By combining this transgenic approach with human PCLD tissue we have shown that via TGFβ-SMAD signalling, cystic BECs condition their microenvironment to promote integrin-dependent cyst-growth. Of note, recent multimodal single nuclear RNA and ATAC sequencing studies in patients with autosomal dominant polycystic kidney disease identified a TGFβ/SMAD3 signature across a number of cell types, including within cystic epithelial cells (59). Collectively, these data suggest that although the cell or tissue of origin of cysts is different and there is diversity in the genetic drivers of polycystic disease, the cellular processes used by epithelial cells to become cystic share common features and potentially common pharmacologically actionable targets.

Polycystic diseases are typified by the production of many, often adjacent cysts within a tissue. How this pattern of disease arises, however, is contentious and even if all cells within a cyst are derived from a single clonal event following loss of heterozygosity (as has been suggested(53)), this would not necessarily account for the multitude of anatomically distinct cysts that form throughout a patient’s lifetime. How cystic tissue undergoes dynamic cellular remodelling to form a polycystic pattern remains unknown, is this a process reminiscent of intestinal crypt fission, where inflation and collapse result in new crypt structures commonly found in pre-neoplastic colonic disease (60–62), or pancreatic regeneration where acini divide to promote functional repair (63). Using multispectral lineage tracing *in vivo*, our data demonstrate that rather than cysts forming and growing as either ever-expanding structures or forming polycystic disease patterns as part of a passive process of inflation and collapse, dynamic septa are formed within cysts which drives structural cystic division by reactivation of tissue morphogenesis and cytoskeletal remodelling. Furthermore, our data indicate that the pattern of disease seen in PCLD, where many cysts from in close proximity, is generated by the division of pre-existing cysts rather than single large cysts expanding. Cyst fission therefore reveals potential pharmacological targets that can be leveraged to limit the formation of cysts across heterogeneous genetic causes.

There are limitations to this study. PCLD evolves throughout a patient’s lifetime and although we demonstrate that cyst generation is an ongoing process with active remodelling of the biliary epithelium, we are limited in our understanding of cyst-initiating mechanisms and our current study cannot address whether there are earlier opportunities to limit the transformation of healthy tissue into cysts. Moreover, although we have concentrated on the epithelial component of cysts, there are likely a variety of stromal and immune cells that support cystic growth and cyst fission. We did not assess the importance of these in this work and to do so will be essential to build a complete picture of the cystic process. Last, *Wdr35*-loss is simply a model of liver cyst formation and within patients there have been a suite of causative mutations identified. Some of these are essential cilia genes (including *WDR35)*, however the most commonly mutated proteins constitute the polycystin complex and many others are involved in protein trafficking and processing within the endoplasmic reticulum. Although we have shown that across a range of genetic mutations common cellular processes are activated, without systematic deletion of these “cystic” genes we are unable to comment on the diverse features that each mutation could infer.

## Materials and Methods

### Study design

The primary hypothesis of this study was that loss of the primary cilium results in global changes to cholangiocyte signalling to disrupt bile duct homeostasis. Secondary hypotheses regarding the positive roles of TGFβ and integrin signalling in cyst growth were developed as a consequence of the first hypothesis being proven true and tested using pharmacological and genetic inhibition studies. The sample size of this study was not defined *a priori* and sample sizes are provided within the figure legends. In experiments where animals or cultures received inhibitors, these were randomized prior to dosing. All animal tissues were collected blinded and analysed without knowledge of to which group they belonged. Complete materials and methods can be found within the supplementary materials.

### Human tissue and animals studies

JPH Drenth (Department of Gastroenterology and Hepatology, Institute for Molecular Life Sciences, Radboud University Medical Center, NL) provided samples from patients with PCLD with known genetics under ethical approval 2012/317. Further samples were provided by the NHS Lothian and University of Edinburgh Academic and Clinical Central Office for Research & Development (ACCORD) biobank under ethical code TGU-LAB-1512. All tissues were collected under informed consent or were archival diagnostic specimens. Human tissue details are summarised in Data file S7.

Animals were maintained in SPF environment and studies carried out in accordance with the guidance issued by the Medical Research Council in “Responsibility in the Use of Animals in Medical Research” (July 1993) and licensed by the Home Office under the Animals (Scientific Procedures) Act 1986. Experiments were performed under project license number PFD31D3D4 in facilities at the University of Edinburgh (PEL 60/6025).

### Statistical analysis

All experimental groups were analysed for normality using a D’Agostino–Pearson Omnibus test. Groups that were normally distributed were compared with either a two-tailed Student’s t test or a one-way ANOVA with a post hoc correction for multiple testing. Non-parametric data were analysed using a Wilcoxon–Mann–Whitney U test or a Kruskall–Wallis test when comparing multiple non-parametric data. Throughout, P< 0.05 was considered significant. Data are represented as mean with S.E.M. for parametric data or median with S.D. for non-parametric data. All n= are individual animals and N= are technical or experimental replicates and are and defined in the figure legends.

All figures were assembled with Adobe Illustrator and graphics were created with BioRender.com.

## Supplementary Material

Supplementary Material

## Data Availability

All data is available in the manuscript or the supplementary materials. RNAseq data from this study is available from Dryad (doi:10.5061/dryad.mkkwh7152) and from NCBI GEO accession GSE145271. All materials generated as part of this study will be made available upon request to the corresponding authors.

## One sentence summary

TGFβ-ECM-Integrin signalling promotes polycystic liver disease by driving cyst growth and fission, which can be pharmacologically inhibited.
