# Supplementary material for: A TGFβ-ECM-Integrin signalling axis drives structural reconfiguration of the bile duct to promote polycystic liver disease
Source: Sci Transl Med. Author manuscript; Available in PMC 2023 Oct 25. (PMC7615241; doi:10.1126/scitranslmed.abq5930)
Supplement: Supplementary Material [file EMS189123-supplement-Supplementary_Material.pdf]

A TGFβ-ECM-Integrin signalling axis drives structural reconfiguration of the bile duct to promote polycystic liver disease.

Scott H Waddell, Yuelin Yao, Paula Olaizola, Alexander Walker, Edward J Jarman, Konstantinos Gournopanos, Andreea Gradinaru, Ersi Christodoulou, Philippe Gautier, Melissa M Boerrigter, Massimiliano Cadamuro, Luca Fabris, Joost PH Drenth, Timothy J Kendall, Jesus M Banales, Ava Khamseh, Pleasantine Mill, Luke Boulter

Supplementary Materials and Methods

Figure S1: Loss of Wdr35 in BECs promotes increased cyst formation over time.
Figure S2: Cyst formation in Wdr35-/- animals demonstrates sexual dimorphism seen in human PCLD patients.
Figure S3: The loss of cilia in murine BECS in adult animals does not universally affect liver function.
Figure S4: Normal and cystic BECs can be isolated from the liver using EpCAM.
Figure S5: Cystic BECs are transcriptionally distinct from normal BECs.
Figure S6: Cystic BECs are typified by a distinct transcriptional programme and have specific markers.
Figure S7: Cystic BECs have specific protein marker expression.
Figure S8: BEC markers are consistently expressed across normal and cystic cells.
Figure S9: Cystic BECs are enriched for components of Ca2+ and MAPK signalling.
Figure S10: Cystic BECs in mice have high TGFβ pathway expression and activity.
Figure S11: Human BECs treated with recombinant TGFβ increase ECM expression
Figure S12: Human cholangiocytes are sensitive to treatment with SIS3.
Figure S13: Mouse cystic BECs are enriched for extracellular matrix transcripts.
Figure S14: Blood biochemistry from mice treated with the SMAD3 inhibitor, SIS3.
Figure S15: Cystic BECs express a novel profile of integrin molecules.
Figure S16: The formation of a pro-cystic state driven by integrin-α2β1 is common across genetic subtypes of PCLD.
Figure S17: PCLD forms through the structural fission of pre-existing cysts.
Figure S18: Examples of cystic fission using multi-colour lineage tracing.
Figure S19: Human cells are sensitive to treatment with TC-I 15.
Figure S20: Integrin-α2β1 inhibition reduces ITGA2 expression and alters blood biochemistry.
Figure S21: A schematic model demonstrating how liver cysts grow.

Table S1: Differentially expressed genes between cluster 1 and 2 following single cell analysis.
Table S2: Enriched GO and KEGG terms when comparing scRNA from cluster 1 and 2.
Table S3: Differentially expressed genes between vehicle and SIS3 treated cyst-bearing animals.
Table S4: GOrilla and REViGO outputs from DEGs presented in Table S3.
Table S5: Putative cell autonomous ligand-receptor interactions in wild-type epithelial cells.
Table S6: Putative cell autonomous ligand-receptor interactions in cystic epithelial cells.
Table S7: Clinical characteristics of human tissue samples.
Table S8: Antibodies used in this study.

Materials and Methods

Animal models: Transgenic deletion of *Wdr35*: Mice carrying floxed alleles of exon 2 of *Wdr35* (*Wdr35*$^{flox/flox}$)(64) were crossed with animals expressing *Keratin19-CreER*^{T}(65). Animals were bred such that all mice contained *CreER*^{T}, experimental animals were homozygous for the *Wdr35*$^{flox/flox}$ allele and controls were wild type (*Wdr35*$^{+/+}$). *K19CreER*^{T}*;Wdr35*$^{flox/flox}$ or *K19CreER*^{T}*;Wdr35*$^{+/+}$ animals were treated with tamoxifen (three times 4 mg doses at 6-8 weeks old; Tamoxifen dose was not weight adjusted) by IP injection or oral gavage to activate CRE. Tamoxifen was dissolved in 5% molecular grade ethanol:95% corn oil. All experiments were run with contemporaneous control groups, such that we accommodated for route of tamoxifen administration. Administration of tamoxifen by oral gavage limited tamoxifen induced-toxicity and induced higher cyst formation (**Fig. S2**). After tamoxifen treatment, transgenic animals that have lost *Wdr35* are denoted as *Wdr35*$^{-/-}$. Animals were housed in same sex groups in 12 h light-dark cycles, with access to food and water *ad libitum*. Both males and females were used in this study. For experiments containing the *R26*$^{LSL\text{-}Confetti}$ reporter(66), *K19CreER*^{T}*;Wdr35*$^{flox/flox}$ were crossed to generate *K19CreER*^{T}*;Wdr35*$^{flox/flox}$*; R26*$^{LSL\text{-}Confetti/+}$ mice and were treated with tamoxifen as detailed above. For inhibitor studies, cyst-bearing animals were treated for 3 weeks with 20 mg/kg of TC-I 15 (a selective integrin α2β1 inhibitor, which was dissolved in DMSO at 50 mg/ml), 10 mg/kg of SIS3 (an inhibitor of SMAD3, dissolved in DMSO at 25 mg/ml) or vehicle alone. Both dissolved compounds were mixed with corn oil (1 part dissolved compound: 11.5 parts corn oil) and animals were dosed via intra-peritoneal injection three times weekly for 3 weeks. For *K19CreER*^{T}*;Itga2*$^{flox/flox}$ duct cultures, K19CreERT mice were crossed with mice containing a Cre-inducible *tdTomato* (R26R$^{LSL\text{-}tdTomato}$, Jax: 007914)(67) and *Itga2*$^{flox/flox}$ (Jax: 018921)(68) alleles.

Animals were maintained in SPF environment and studies carried out in accordance with the guidance issued by the Medical Research Council in "Responsibility in the Use of Animals in Medical Research" (July 1993) and licensed by the Home Office under the Animals (Scientific Procedures) Act 1986.

Experiments were performed under project license number PFD31D3D4 in facilities at the University of Edinburgh (PEL 60/6025).

Blood biochemistry: At necropsy, blood was collected from the inferior vena cava and spun at 12,000 g to pellet the cellular fraction. Serum was collected and provided to the SURF facility (Queens Medical Research Institute, Edinburgh) where they performed ELISA for aspartate transaminase, alanine aminotransferase, glutamate dehydrogenase, albumin, bilirubin, and urea.

Fluorescent activated cell sorting and 10X single cell sequencing: 12 month *K19CreER^T; Wdr35$^{-/-}$* or *K19CreER^T; Wdr35$^{+/+}$* mice were perfused with saline and livers were digested with collagenase and dispase to enrich for the biliary tree. Enriched bile ducts were dissociated into single cells using trypsin and stained for EpCAM-APC, CD31-PECy7, CD45-PECy7 (**Table S8**). Live cells (which are negative for DAPI) were identified and CD31-PECy7-/CD45-PECy7-/EpCAM+ cells were isolated and 20,000 cells were used as input for Chromium Next GEM Single Cell 3' analysis by the FACS and Single Cell Core (MRC Human Genetics Unit, Edinburgh). Generated libraries were quantified and sequenced on an Illumina P1000 flow cell by the Wellcome Trust Clinical Research Facility (University of Edinburgh). All RNA sequencing data pertaining to this manuscript is deposited on Dryad (https://doi.org/10.5061/dryad.mkkwh7152). Bulk RNA data from TGFβ-treated H69 cells was downloaded from GSE145271.

Analysis of single cell RNAseq data: Raw sequencing data was analysed using Cell Ranger's pipelines (version 5.0.0) provided by 10x Genomics. Specifically, 'cellranger mkfastq' was initially used to generate FASTQ files from raw base call files. Then, 'cellranger count' was applied to align FASTQ files to the pre-build mouse reference mouse genome (GRCm38/mm10) provided by Cell Ranger and generate single cell feature counts.

Seurat (v4.0.6, https://satijalab.org/seurat/) was used to filter out cells: (1) with unique feature counts over 7,000 or less than 600, (2) with counts greater than 50,000, (3) with more than 5% mitochondrial counts, (4) without expression of BEC marker *Epcam* and *Spp1*. DoubletFinder (v2.0) was used to

predict and remove technical artefacts or 'doublets' for each sample. Specifically, estimated doublet rates were calculated according to the 10x Chromium User Guide. Homotypic doublet rates were also taken into account using the modelHomotypic function with default parameters. We subsequently removed genes that are present in less than 3 cells in each sample.

After the pre-processing and quality filtering steps, the dataset was then processed using the standard Seurat workflow: 1. log normalize data using the NormalizeData function, with a default size factor 10,000; 2. scale data using the ScaleData function; 3. identify variable features using FindVariableFeatures with "vst" parameters and then imputed into PCA using RunPCA function. RunUMAP function was used for UMAP embedding visualisation with the first 10 PCs (principal components).

Ward-linkage hierarchical clustering was performed with squared Euclidean distance on the first 10 PCs to obtain clusters (arXiv: 2012.02936v2 [stat.ME]). Before proceeding to downstream analyses, such as differential gene expression analysis, it is important to avoid the problem of selective inference. Selective inference is described as the assessment of significance as well as effect sizes from the same dataset which has been carried out via statistical tests to find potential associations. Selective inference has recently been demonstrated to have an important role in accurately estimating significance in biomedical science –omic data(69). Specifically, in the scRNA-seq clustering analysis, one first clusters the data, then measures if they are significantly different and identifies differentially expressed genes between clusters. This leads to inflated Type I error rate (artificially deflated p-values). We therefore applied selective inference correction implemented by clusterpval R (arXiv: 2012.02936v2 [stat.ME]) package to determine an appropriate number of clusters and confirmed that the identified clusters are pair-wise significantly different. We also assessed the stability of estimated clusters under different PC projections. We then used FindMarkers function from Seurat to identify differentially expressed genes (DEGs) whose log-fold changes are greater than 0.25, BH-adjusted p-values less than 0.05 by the Wilcox test and are expressed by at least 25% of cells.

The clusterProfiler package was used to perform Gene Set Enrichment Analysis for DEGs. The enrichKEGG and enrichGO function were used to identify significant GO (Gene Ontology) terms and KEGG (Kyoto Encyclopaedia of Genes and Genomes), respectively, with threshold p value < 0.05 and q value < 0.05. The background gene set of this analysis was selected from publication literature(70), which includes a list of genes that could be expressed in the *EpCam* sorted cholangiocytes from mouse. R package ggplot2 was used to visualise the results.

We performed cellular trajectory analysis by RNA velocity. We first used velocyto (v.0.17)(71) to generate the loom files from pre-aligned bam files. The loom files contain two count matrices of unspliced and spliced RNA abundances.

The proportions of spliced and unspliced counts are 80% and 20% respectively, which is in the appropriate range of velocity inference(72). We then calculated the RNA velocity from scVelo(72) using deterministic (steady-state), stochastic and dynamical mode. They produced consistent results.

Receptor-ligand analysis was performed using R package SingleCellSingalR(73). Gene expression data and hierarchical clustering were used as input to compute receptor-ligand interactions scores between clusters using the 'cell_signaling' function. We filtered out receptor-ligand interactions whose LRscores were lower than 0.5. Receptor-ligand interactions were visualized by the 'visualize_interactions' function.

Isolation of RNA and DNA: RNA was extracted from FACS isolated cells and extracted by TRIzol RNA Isolation Reagent (Invitrogen) lysis. RNA was precipitated with chloroform and cleaned up using the RNeasy Mini Kit (QIAGEN) as per the manufacturer's instructions. For downstream sequencing applications RNA quality (RIN score) was quantified using the Agilent 2100 Bioanalyzer with an RNA 6000 chip. A minimum RIN threshold of 8 was used for RNA-seq.

RNA Sequencing: Total-RNA samples were fragmented to a size appropriate for sequencing on an Illumina platform and first-strand cDNA was generated using the SMARTer Stranded Total RNA-Seq

Kit v2 – Pico Input Mammalian kit (Clontech Laboratories, Inc. #634411). Illumina-compatible adapters and indexes were then added via 5 cycles of PCR. Depletion of ribosomal cDNA (cDNA fragments originating from highly abundant rRNA molecules) was performed using ZapR v2 and R-probes v2 specific to mammalian ribosomal RNA and human mitochondrial rRNA. Sequencing was performed on the NextSeq 2000 platform (Illumina Inc, #20038897) using NextSeq 2000 P2 Reagents (200 Cycles) (#20046812). Libraries were combined in a single equimolar pool of nine based on Qubit and Bioanalyser assay results and run on a single P2 flow cell. PhiX Control v3 (Illumina, #FC-110-3001) was spiked in at 1% library concentration to facilitate troubleshooting in the event of any run issues.

RNA sequencing data processing and analysis: The primary RNA-Seq processing, quality control to transcript-level quantitation, was carried out using nf-core/rnaseq v1.4.3dev (https://github.com/ameynert/rnaseq)(74). Reads were mapped to the mouse FVB_NJ_v1 decoy-aware transcriptome using the salmon aligner (1.1.0). RNA-Seq analysis was performed in R (4.0.2), Reads were summarized to gene-level and differential expression analysis was performed using the bioconductor packages tximport (1.16.1) and DESeq2 (1.28.1). A pre-filtering was applied to keep only genes that have at least 10 reads in a group and 15 reads in total. The Wald test was used for hypothesis testing for pairwise group analysis. A shrunken log2 fold changes (LFC) was also computed for each comparison using the adaptive shrinkage estimator from the 'ashr' package. Gene expression changes in H69 cells in response to TGFβ1 treatment were assessed using the public available dataset from GSE145271.

Duct isolation and culture: Livers were minced finely using razor blades and digested with 0.125 mg/ml collagenase type IV (Gibco) and 0.125 mg/ml dispase II (Gibco) at 37 °C. Once bile ducts were visible they were used for FACS, immunostaining or organoid cultures. For cultures, isolated bile ducts were suspended in Matrigel (Corning) and cultured in media containing Advanced DMEM/F-12 media (Gibco) containing 1x GlutaMAX (Gibco), 1x Antibiotic-Antimycotic (Gibco), 10 µM HEPES (Sigma), 50 ng/ml EGF (R&D Systems), 100 ng/ml FGF10 (Novus Biologicals), 5 ng/ml HGF (Novus Biologicals), 10

nM gastrin (Sigma), 10 µM nicotinamide (Acros Organics), 1.25 mM N-acetyl-Lcysteine (Sigma), 1x B27 (Life Technologies), 1x N2 Supplement (Life Technologies), 1 µg/ml R-Spondin-1 (R&D Systems), 0.2 µg/ml WNT5A (R&D Systems) and 10 nM forskolin (Tocris). Cysts were allowed to culture for 72 hours at 37 °C in a humidified incubator with 5% CO2 before fixing for immunofluorescent staining or lysis for proteomic studies. Inhibition studies had media supplemented with 100 µM TC-I 15 (integrin-α2β1 selective inhibitor), 10 µM SIS3 (SMAD3 selective inhibitor) or DMSO (vehicle control) at equivalent volumes. Media containing inhibitors/vehicle was replaced after 48 hours. Cyst size was determined by brightfield microscopy and measurements made on Fiji (ImageJ).

Cell culture: Human (normal human cholangiocytes [NHC], H69 cells, ADPLD and ADPKD) cholangiocyte cells were seeded on collagen-coated flasks and cultured in supplemented(75) DMEM/F-12 medium as previously described(76). Cell proliferation rates were determined by flow-cytometry using CellTrace CFSE Cell Proliferation Kit (Invitrogen), FITC Annexin V (Biolegend) with TO-PRO-3 Iodide (Invitrogen) were used to quantify proliferation and apoptosis, respectively, and Cell Proliferation Reagent WST-1 (ROCHE) was used to measure cell viability following the manufacturers' instructions for each. The small molecule inhibitors TC-I 15 and SIS3 were dissolved in DMSO and cultures were treated at the concentrations stated in the figures for 72h.

Protein Expression: Protein lysates were obtained from tissues using 1% n-Dodecyl-beta-Maltoside Detergent (Thermo Scientific) in dH_2O with 1 cOmplete mini EDTA-free protease inhibitor tablet (Roche) and 1 phosphoSTOP phosphatase inhibitor tablet (Roche) for Western blots; or RPPA lysis buffer (2.5 ml Triton-X-100, 25 ml 0.5 M HEPES pH 7.4, 0.5 ml 0.5 M EGTA pH 7.5-8.0, 37.5 ml 1 M sodium chloride, 0.375 ml 1M magnesium chloride, 0.1 ml 100 mM sodium orthovanadate, 1ml 100 mM tetrasodium pyrophosphate, 1 ml 1M sodium fluoride, 1 cOmplete mini EDTA-free protease inhibitor tablet (Roche), 1 phosphoSTOP phosphatase inhibitor tablet (Roche), 1 ml glycerol and 1.9 ml dH_2O) for Reverse Phase Protein Array (performed by HTPU Microarray Services, University of Edinburgh). Protein quantification was determined using Pierce BCA reagent (Pierce) and quantified

using a multiwell plate reader to measure absorbance at 562 nm with use of Albumin standards provided. For Western blots, lysates were loaded (7.5-20 µg protein) onto a 4-12% NuPAGE Bis-Tris gel (Thermo Fisher). Protein lysates were reduced with NuPAGE LDS sample buffer (4x) and NuPAGE Sample Reducing Agent (10x) prior to running. Gels were run using NuPAGE MOPS SDS Running buffer containing NuPAGE Antioxidant. Proteins were transferred onto PVDF membrane (Amersham) using NuPAGE Transfer buffer. Following transfer membranes were either 5% dried milk (Marvel) in PBST. Membranes were incubated with primary antibodies (**Table S8**) in 5% BSA (Sigma Aldrich) at 4 °C overnight. Following washing with PBST, membranes were incubated with HRP-conjugated secondary antibodies (**Table S8**) in 3% dried milk (Marvel) at room temperature for 1 h. Following washing, signal was developed using ECL (Pierce) and visualised using Amersham ImageQuant 800 (Cytiva). Signal was quantified using either FIJI or Image Studio Lite (LI-COR).

Immunohistochemistry and quantification: Dissected tissues were fixed overnight in formalin at 4 °C, embedded in paraffin and were sectioned at 4 µm. Following antigen retrieval (see Supplementary table 8), tissue sections were incubated with antibodies as detailed in Supplementary table 8. Fluorescently stained tissues were counterstained with DAPI prior to imaging. Colorimetric stains were counterstained with haematoxylin and mounted with DPX. DAB mean measurements were quantified using QuPath (https://qupath.github.io/). *$K19CreER^{T}$;$Wdr35^{-/-}$;$R26^{LSL\text{-}Confetti/+}$* and *$K19CreER^{T}$;$Wdr35^{+/+}$;$R26^{LSL\text{-}Confetti/+}$* samples were sectioned at 200 µm using a Krumdieck Tissue Slicer and fixed for 45 min in formalin and then cleared using FUnGI clearing as previously described(77,78. Histological tissues were scanned using a Nanozoomer, using a Nikon A1R or Leica Stellaris confocal microscope and were analysed using either FIJI, Imaris, or QuPath.

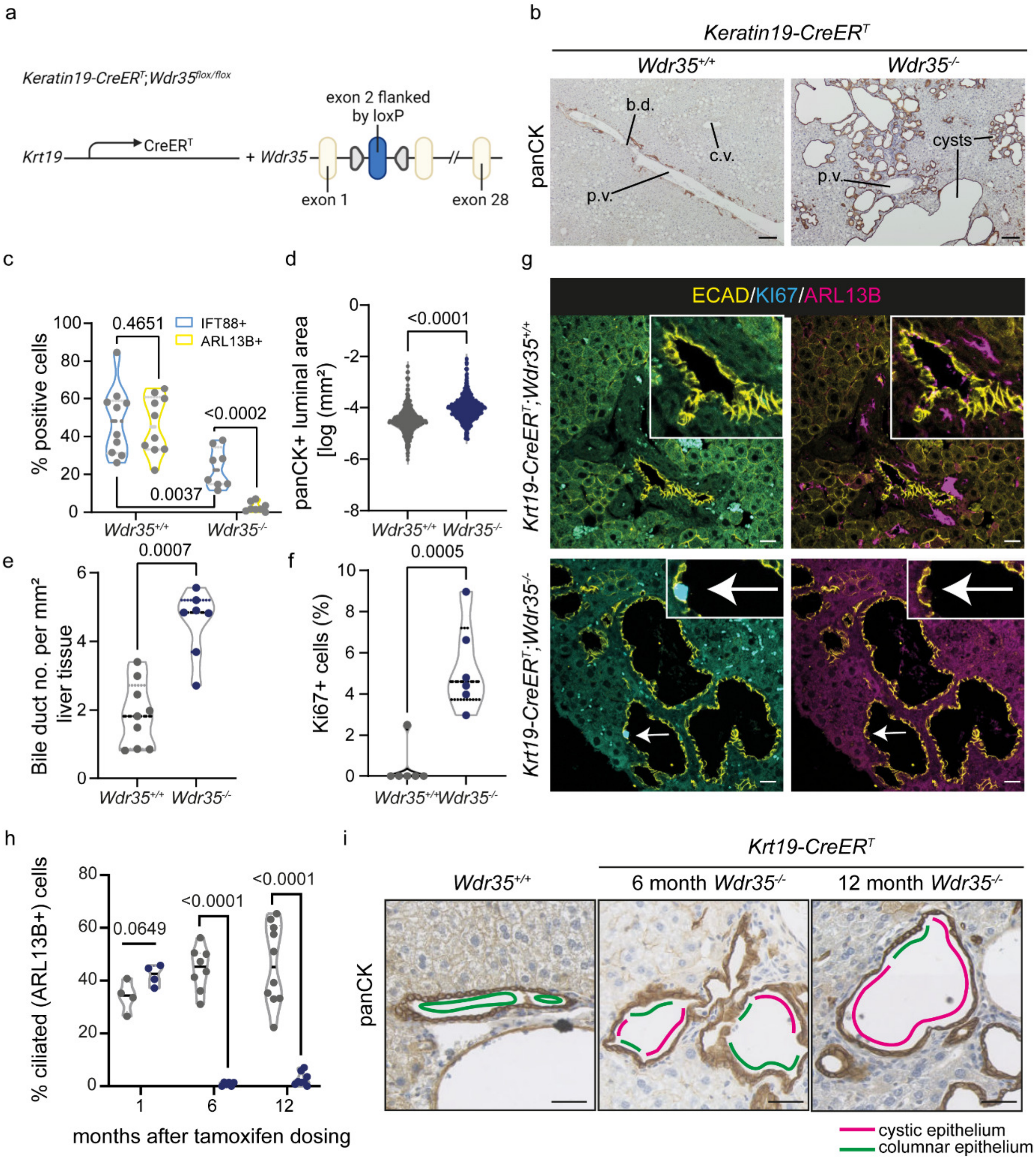

Figure S1: Loss of Wdr35 in BECs promotes increased cyst formation over time. A. Schematic of the genetic strategy for deleting *Wdr35* in biliary cells. **B.** Immunohistochemistry of *Wdr35*$^{+/+}$ and *Wdr35*$^{-/-}$ livers stained for panCK (brown). b.d.-bile duct, c.v.-central vein, p.v.-portal vein **C.** Quantification of IFT88 (cyan) and ARL13B (yellow) positive cilia in *Wdr35*$^{+/+}$ (n=10 mice) and *Wdr35*$^{-/-}$ BECs (n=8 mice). **D.** Luminal area of cysts (*Wdr35*$^{+/+}$ n=426 ducts from 7 mice and *Wdr35*$^{-/-}$ n=659 cysts from 6 mice), and **E.** cyst number per mouse (*Wdr35*$^{+/+}$ n=7 and *Wdr35*$^{-/-}$ n=9), right panel, six months following *Wdr35*-deletion. **F.** Proportion of Ki67 positive (proliferative) cells in cysts 12 months following *Wdr35*-loss (*Wdr35*$^{+/+}$ n=6 animals and *Wdr35*$^{-/-}$ n=6 animals). **G.** Immunohistochemistry showing E-cadherin positive BECs (ECAD, yellow), cilia marker ARL13B (magenta) and proliferation marker KI67 (cyan) in *Wdr35*$^{+/+}$ and *Wdr35*$^{-/-}$ animals. Arrows denote BECs that are positive for KI67. Scale bar = 100µm **H.** number of ARL13B-positive cilia 1, 6 and 12 m following tamoxifen induction in *Wdr35*$^{-/-}$ (blue) animals compared to *Wdr35*$^{+/+}$ (grey). **I.** Immunohistochemistry for panCK on *Wdr35*$^{+/+}$ and *Wdr35*$^{-/-}$ at 6 months (middle panel) and 12 months (right panel). Green lines denote duct regions where cells are columnar and magenta lines are regions occupied by cystic epithelial cells. Scale bar=100 µm.

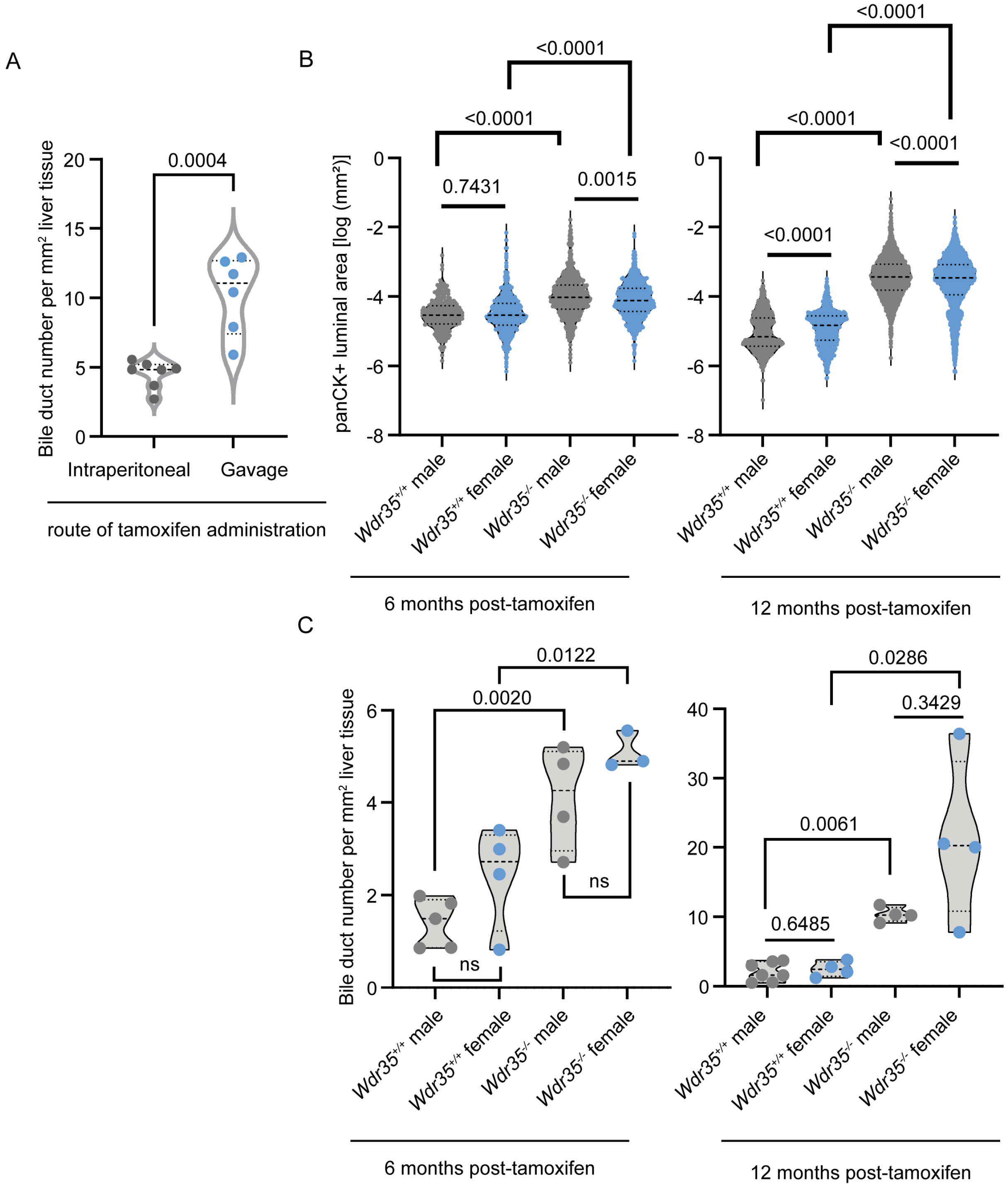

Figure S2: Cyst formation in Wdr35-/- animals demonstrates sexual dimorphism seen in human PCLD patients. A. Route of tamoxifen administration, via intraperitoneal injection (n=7) or via oral gavage (n=6), affects the number of cysts that form within the liver after 6 months. **B.** The area of bile ducts and cysts in both male and female *Wdr35*$^{+/+}$ and *Wdr35*$^{-/-}$ mice 6 m (*Wdr35*$^{+/+}$ male n=286, *Wdr35*$^{+/+}$ female=428, *Wdr35*$^{-/-}$male n=649, *Wdr35*$^{-/-}$ female=581) and 12 m (*Wdr35*$^{+/+}$ male n=642, *Wdr35*$^{+/+}$ female=812, *Wdr35*$^{-/-}$ male n=2380, *Wdr35*$^{flox/flox}$ female=3834) after tamoxifen administration. **C.** Number of bile ducts and cysts in both male and female *Wdr35*$^{+/+}$ and *Wdr35*$^{flox/flox}$ mice 6 m (*Wdr35*$^{+/+}$ male n=5, *Wdr35*$^{+/+}$ female=4, *Wdr35*$^{flox/flox}$ male n=4, *Wdr35*$^{-/-}$ female=3) and 12 m (*Wdr35*$^{+/+}$ male n=7, *Wdr35*$^{+/+}$ female=4, *Wdr35*$^{-/-}$ male n=4, *Wdr35*$^{-/-}$ female=4) after tamoxifen administration.

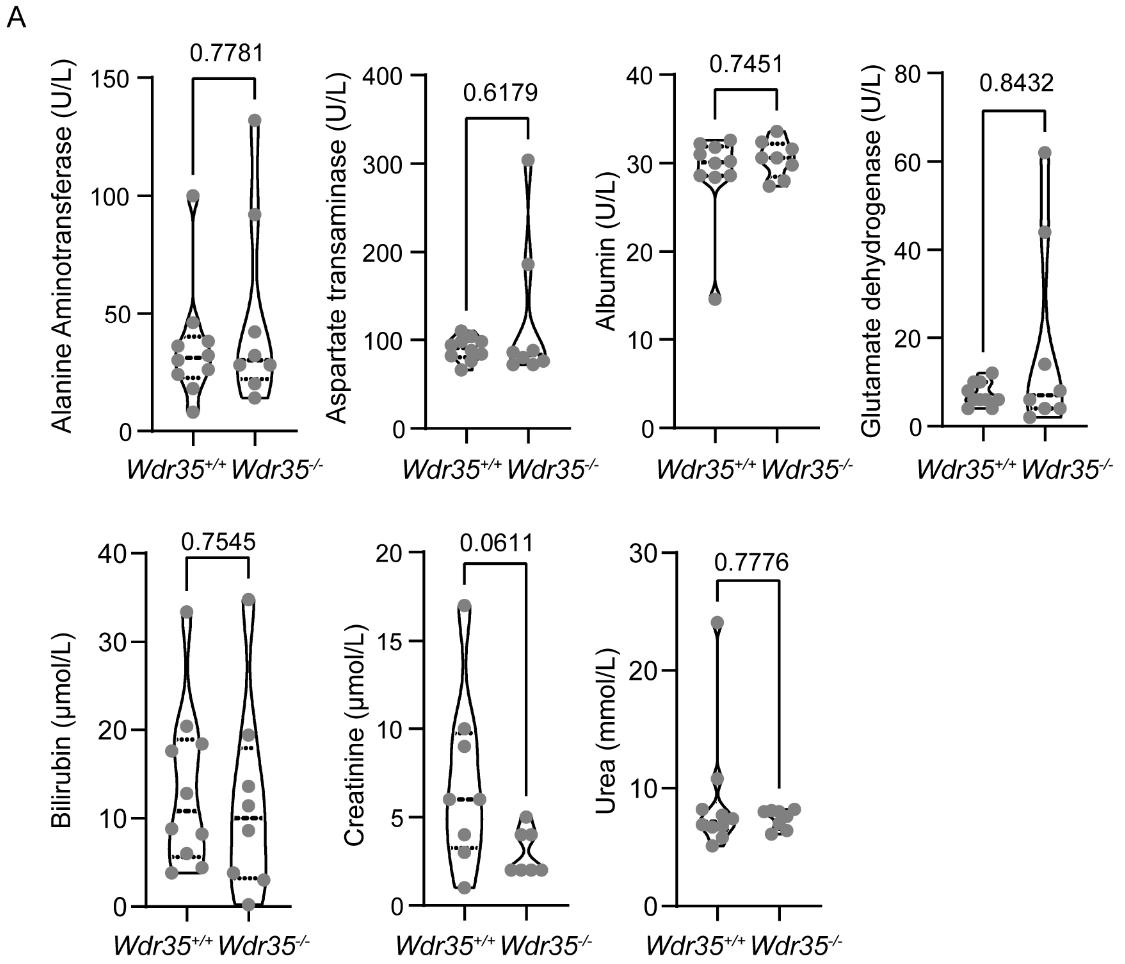

Figure S3: The loss of cilia in murine BECS in adult animals does not universally affect liver function. **A.** Blood serum biochemistry from animals in which *Wdr35* was deleted in BECs 12 months after deletion *Wdr35*$^{+/+}$ (n=10 mice) and *Wdr35*$^{-/-}$ BECs (n=8 mice). See also Figure 1D.

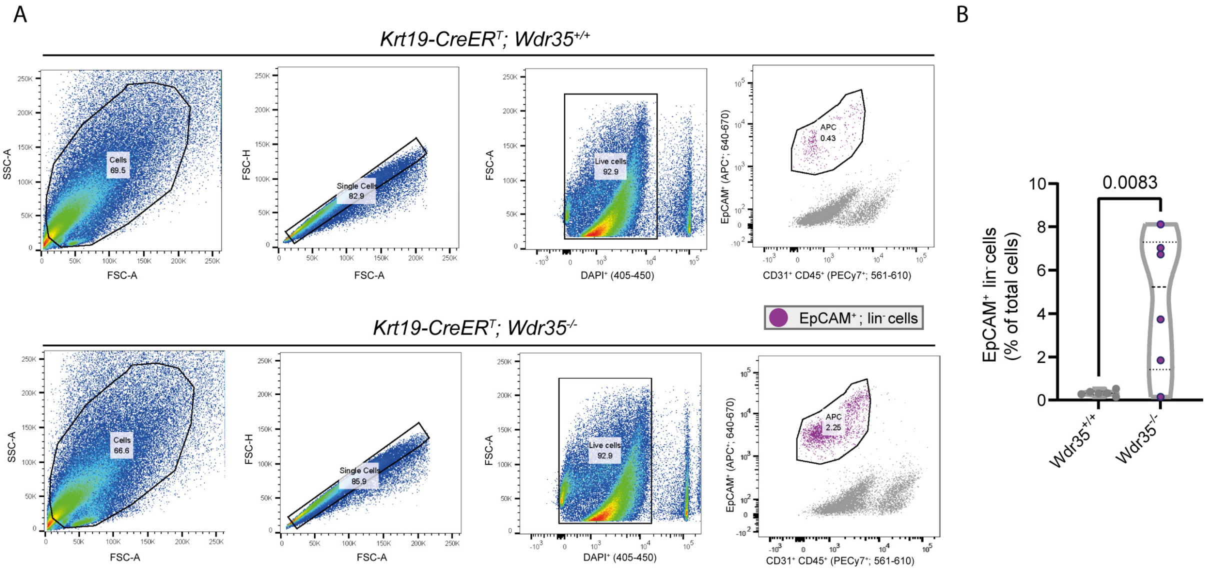

Figure S4: Normal and cystic BECs can be isolated from the liver using EpCAM. A. Flow cytometry gating strategy for single cell sequencing. Briefly, single cells were stained with DAPI. Live cells were then gated for CD45⁻ and CD31⁻ and EpCAM⁺. CD45⁻/CD31⁻/EpCAM⁺ cells were used as input for scRNAseq. **B.** Proportion of total cells that were positive for EpCAM (n=6 mice per genotype)**.**

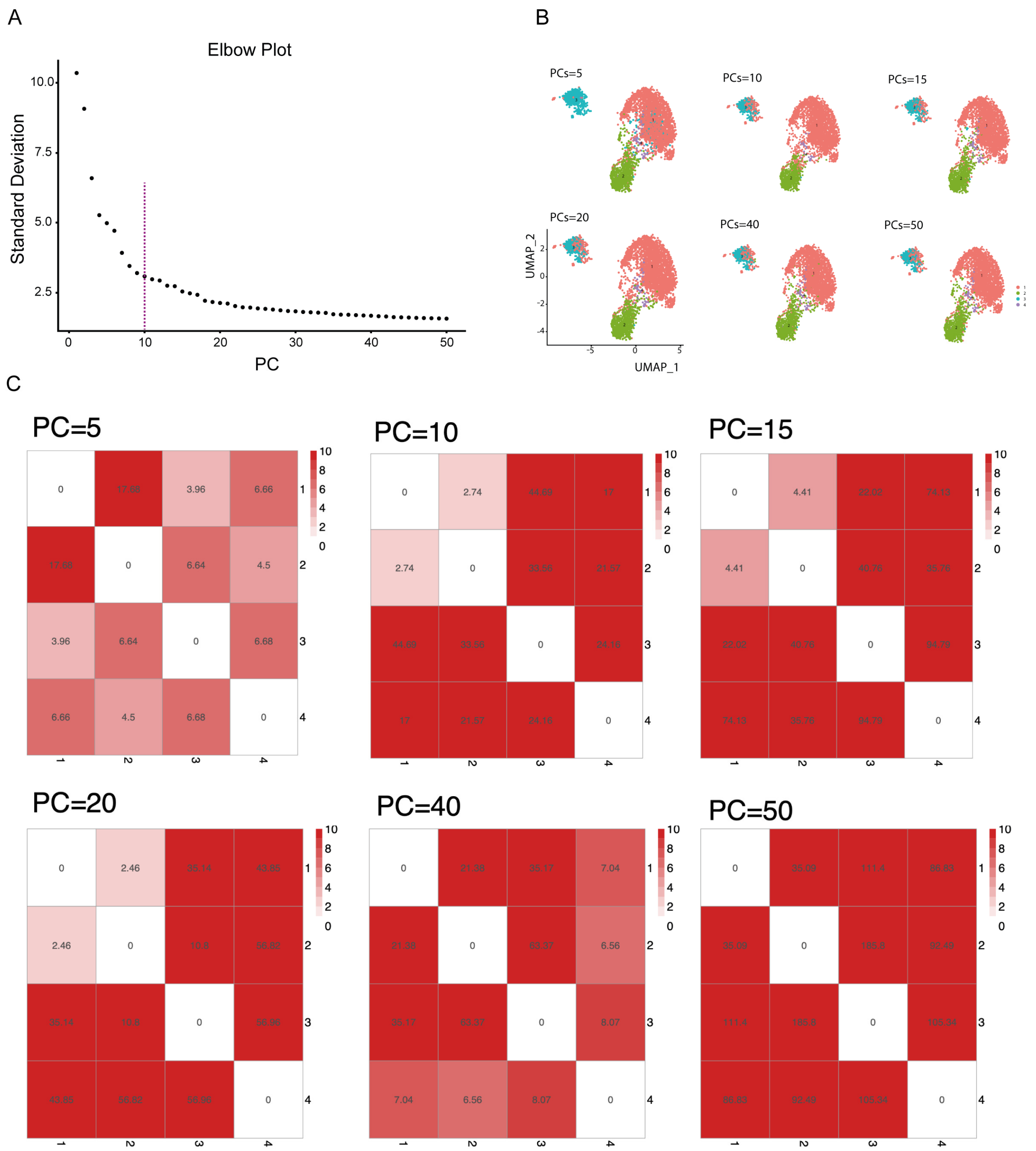

Figure S5: Cystic BECs are transcriptionally distinct from normal BECs. A. The Elbow method was used to determine the optimal number of clusters within the scRNA-seq dataset. The dashed line at 10 Principal Components (PCs) corresponds with the point of inflection. **B.** Ward-linkage hierarchical clustering with squared Euclidean distance was used to obtain 4 clusters and determine cluster stability across different PCs. Increasing from PC5 to PC10 shows a slight difference however beyond PC10 clustering remains stable. **C.** Heatmap showing the $-\log_{10}$ FDR from test difference in mean for all pairs of clusters. The difference between any pairwise clusters was significant. PC10 was determined as the appropriate input for subsequent analyses.

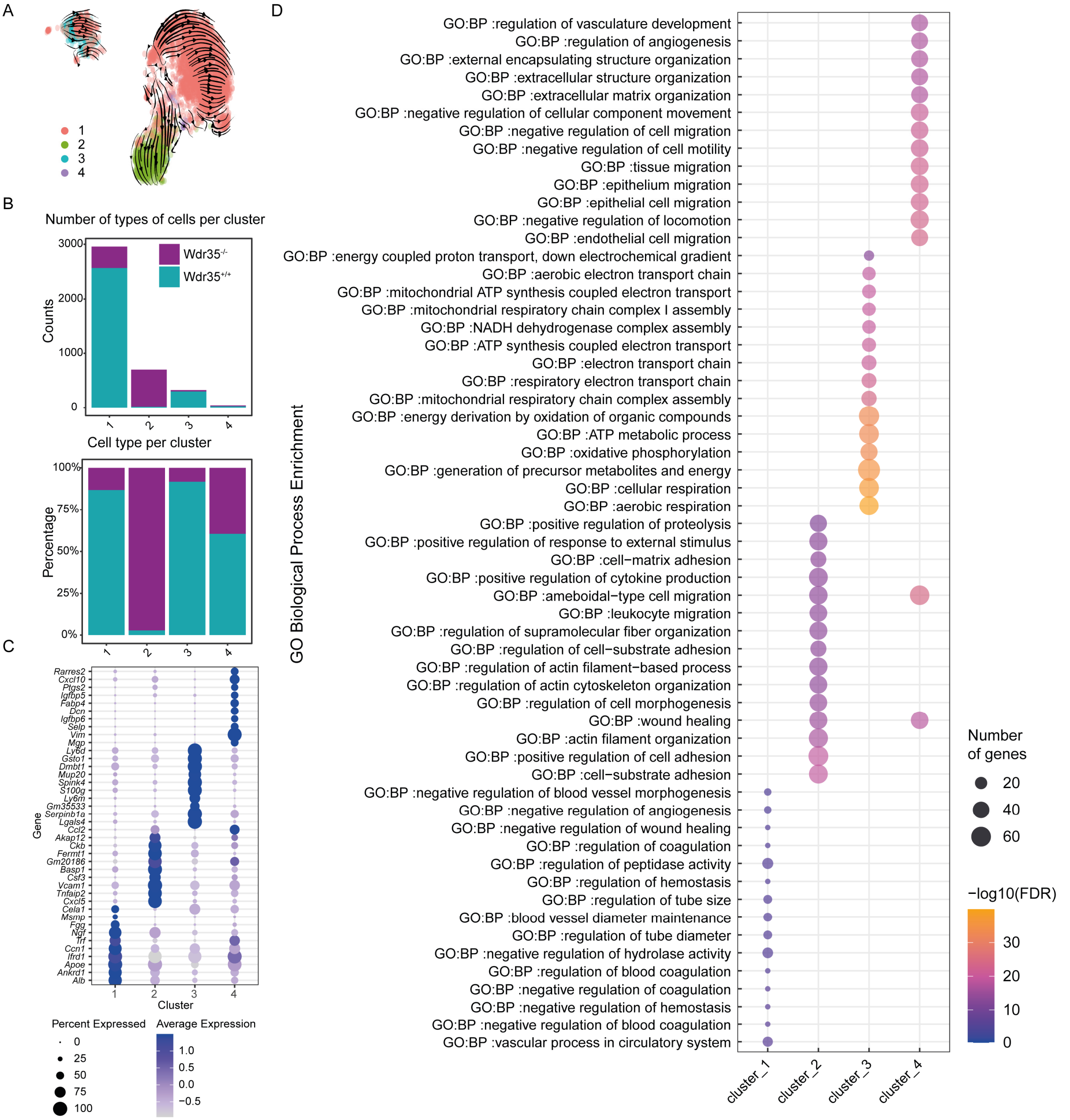

Figure S6: Cystic BECs are typified by a distinct transcriptional programme and have specific markers. A. UMAP embedding of BECs, coloured by identified clusters and overlaid with the RNA velocity stream. **B.** The absolute number of cells from *Wdr35*+/+ and *Wdr35*-/- mutant mice present in each of clusters 1-4 (upper panel) and the proportion of cells from each genotype per cluster (lower panel). **C.** mRNA abundance of the top 10 genes per cluster from cluster 1-4. **D.** Gene Ontology (GO) term demonstrating the Biological Processes that were enriched within each of the four BEC clusters identified from *Wdr35*+/+ and *Wdr35*-/- animals.

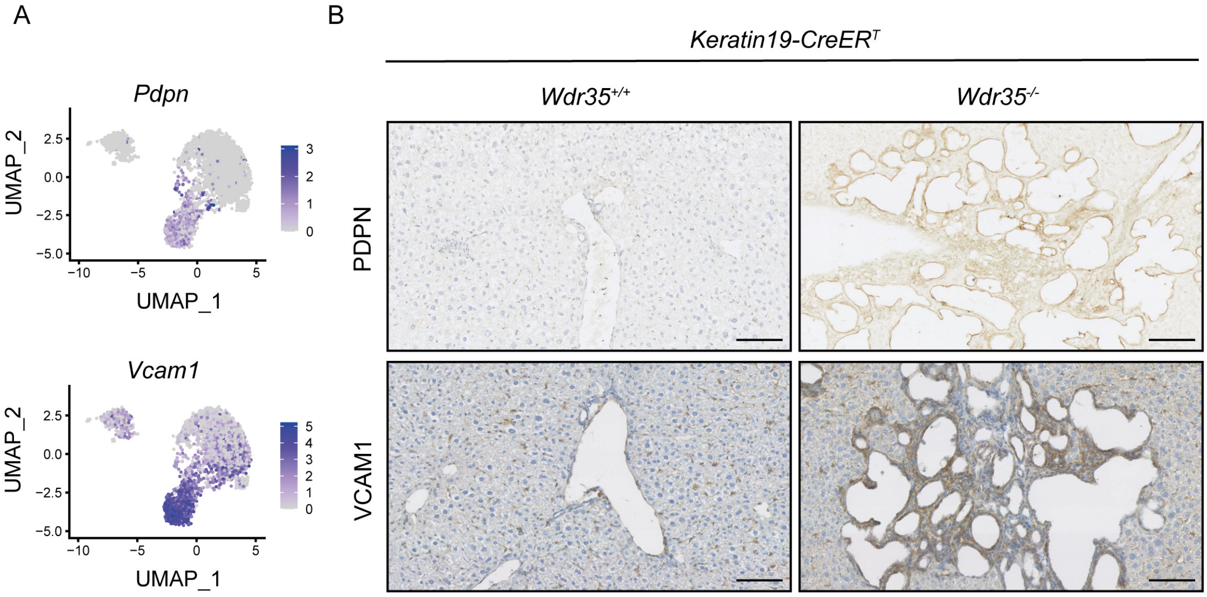

Figure S7: Cystic BECs have specific protein marker expression. A. Transcript expression of *Pdpn* and *Vcam1* specifically in cystic epithelial cells. **B.** Immunohistochemistry for PDPN and VCAM1 in *Wdr35*$^{+/+}$ BECs and *Wdr35*$^{-/-}$ BECs (scale bar=100 µm).

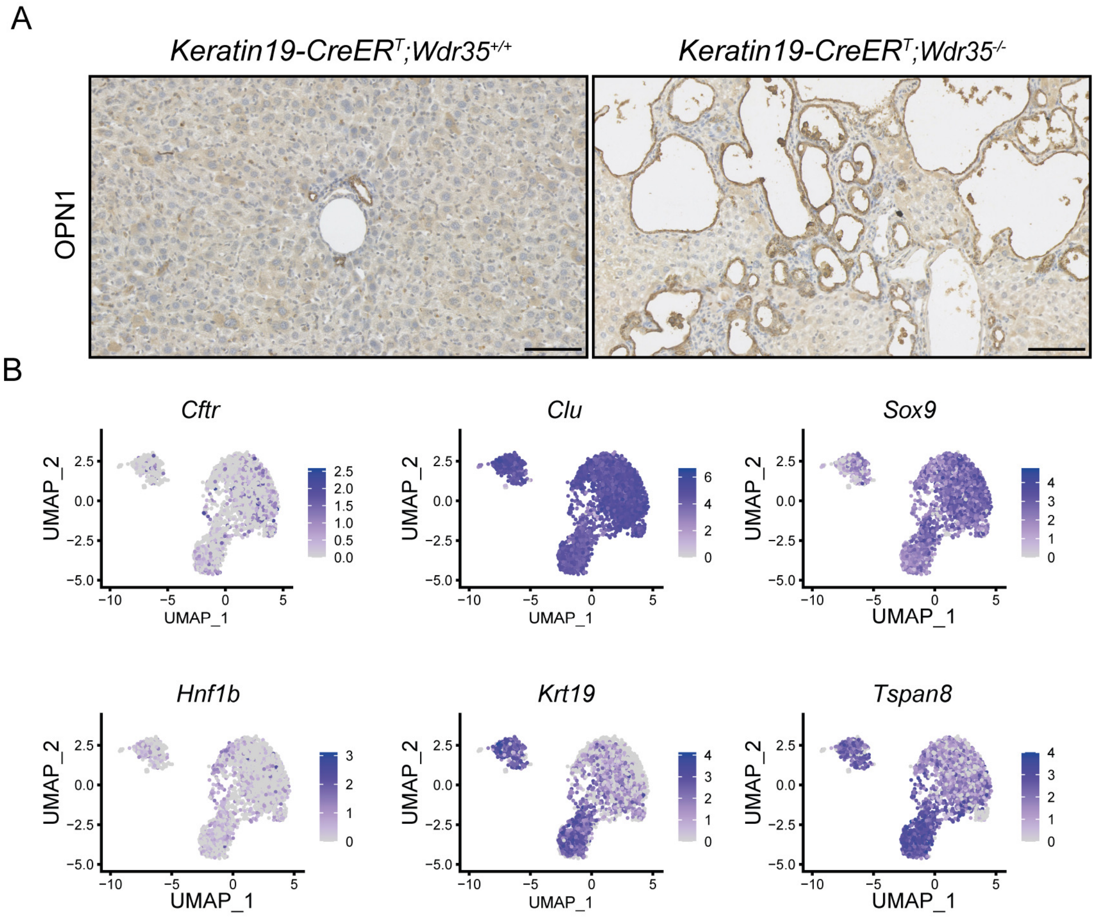

Figure S8: BEC markers are consistently expressed across normal and cystic cells. A. Immunohistochemistry of Osteopontin (OPN1) in both *Wdr35*$^{+/+}$ and *Wdr35*$^{-/-}$ BECs (scale bar=100 µm). **B.** UMAPs showing transcript expression of BEC genes *Cftr*, *Clu*, *Sox9*, *Hnf1β*, *Krt19,* and *Tspan8*.

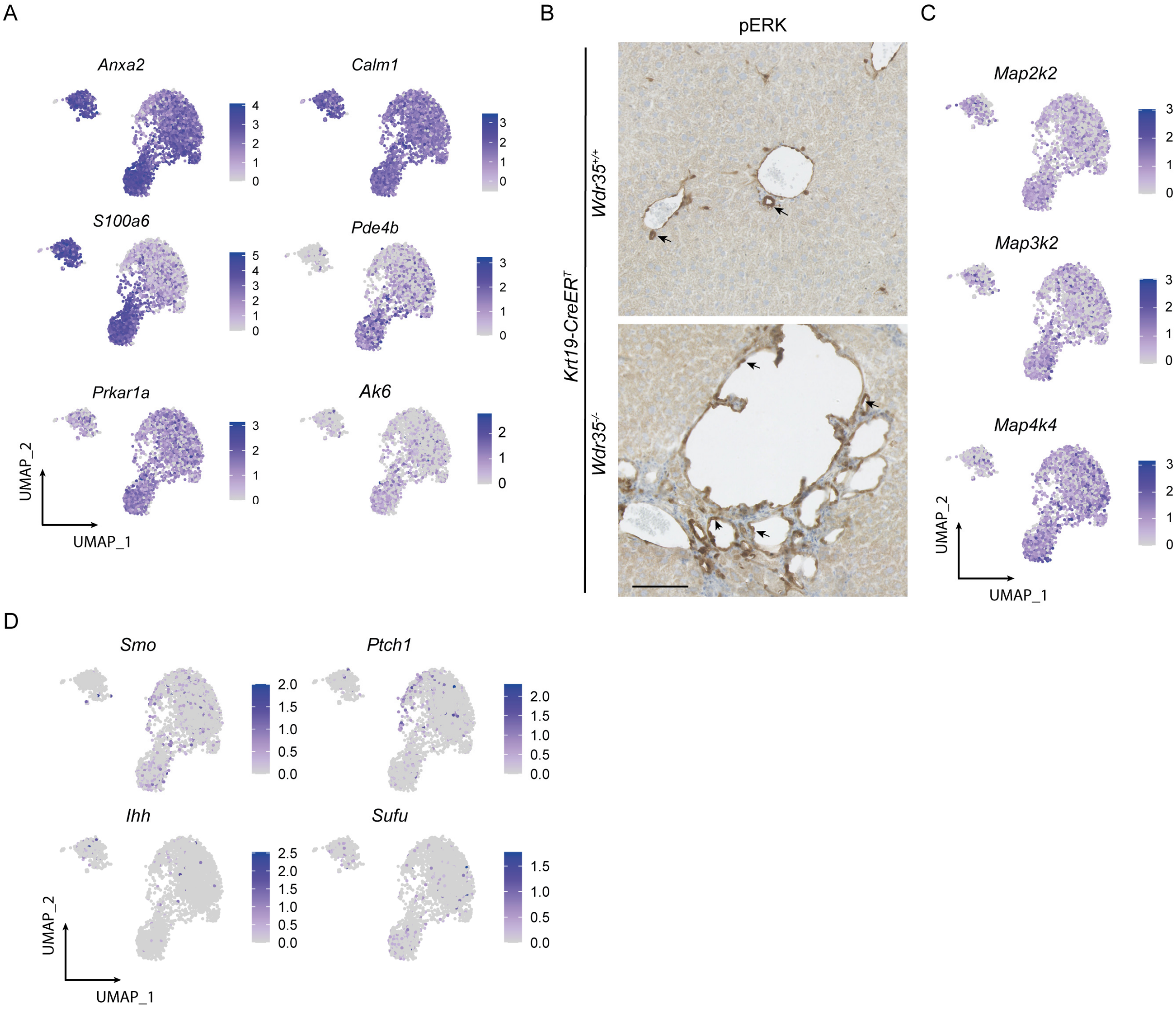

Figure S9: Cystic BECs are enriched for components of Ca^{2+} and MAPK signalling. A. I Transcript expression of Ca^{2+} signalling/PKA components, *Anxa2*, *Calm1*, *S100a6*, *Prkar1a*, *Pde4b,* and *Ak6* is enriched in cystic BECs. **B.** pERK immunohistochemistry *Wdr35*$^{+/+}$ and *Wdr35*$^{-/-}$ ducts arrows denote positive cells. Scale bar = 100 µm. **C.** Transcript expression of MAPK pathway intermediates *Map2k2*, *Map3k2* and *Map4k4* were enriched in cystic BECs. **D.** mRNA expression from scRNAseq of Hedgehog pathway components *Smo*, *Ptch1*, *Ihh*, *Sufu.*

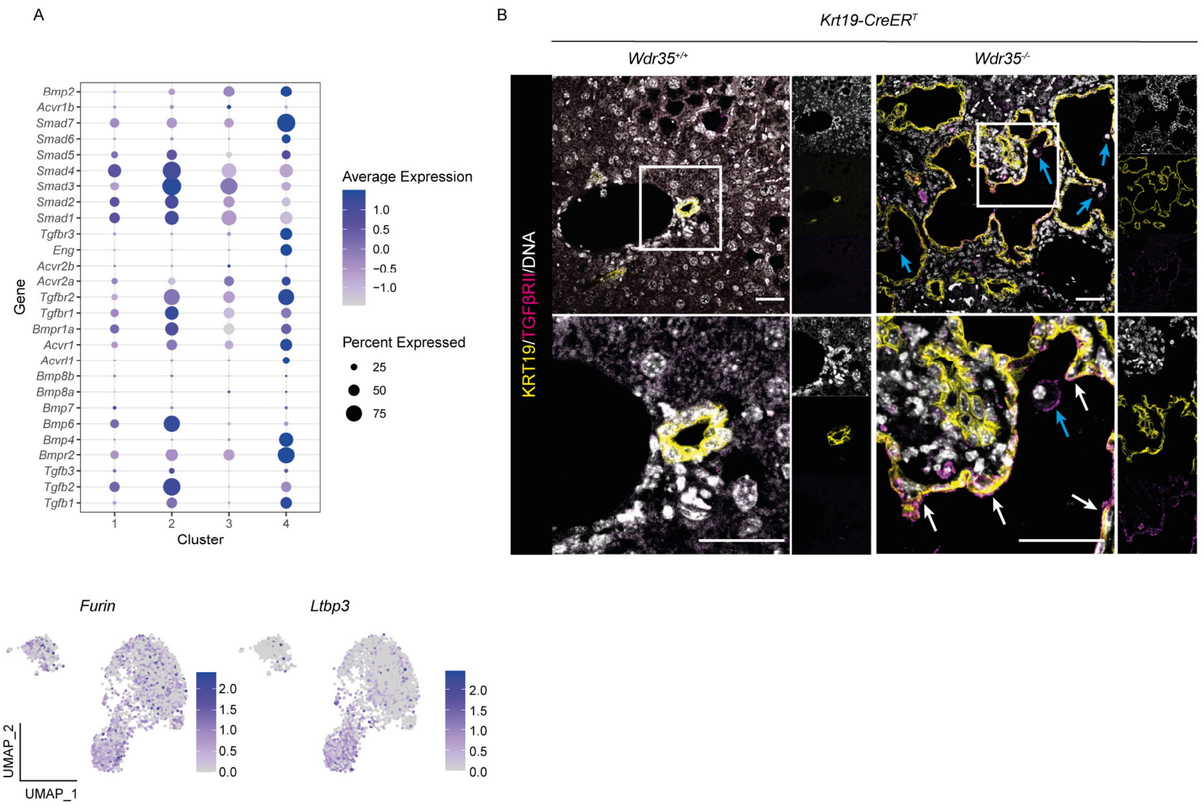

Figure S10: Cystic BECs in mice have high TGFβ pathway expression and activity. A. TGFβ signalling pathway component transcriptional expression across single-cell clusters 1-4 and specific examples of *Furin* and *Ltbp3* which were enriched specifically in cystic cells. **B.** Immunofluorescent staining of *Wdr35*$^{+/+}$ and *Wdr35*$^{-/-}$ liver tissues for KRT19 (yellow) TGFβRII (magenta) and DNA (grey). White arrows denote epithelial positivity, blue arrows positivity on intraluminal immune cells. Scale bar = 50µm.

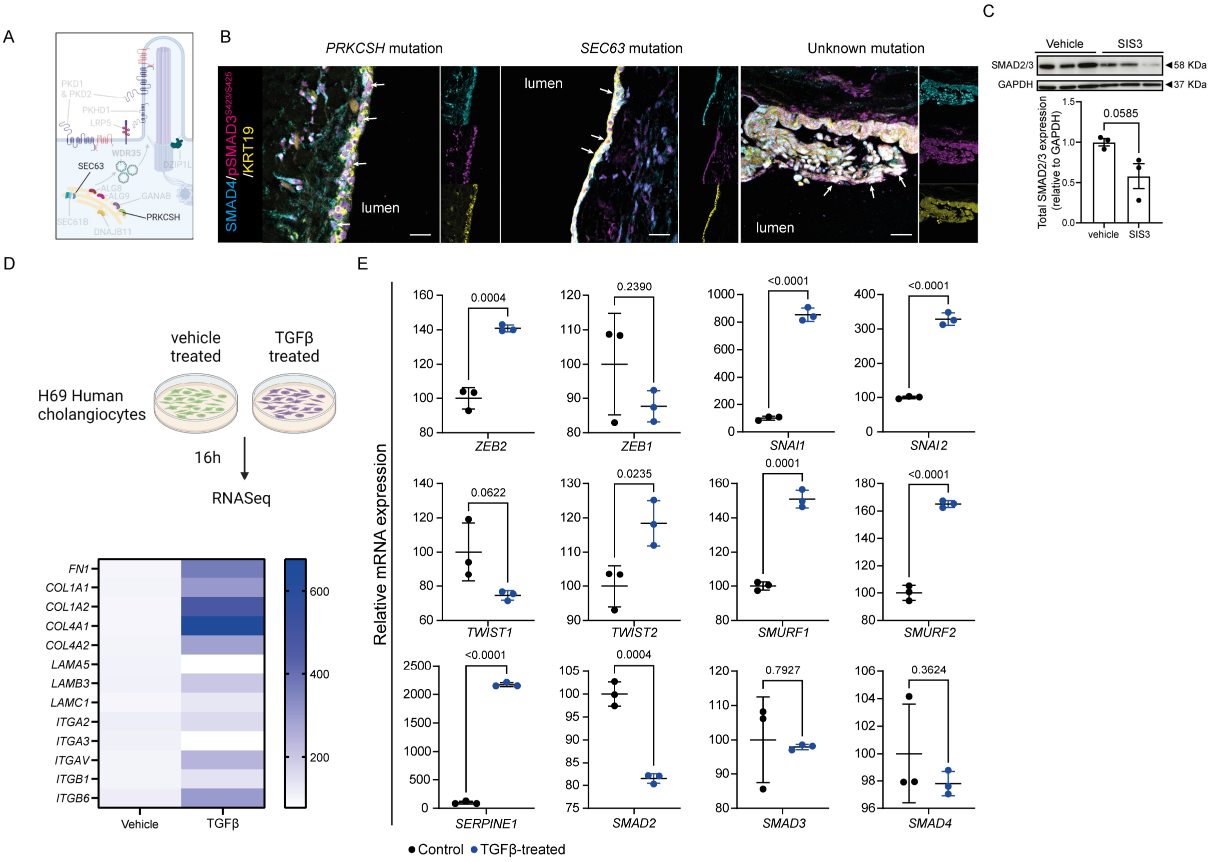

Figure S11: Human BECs treated with recombinant TGFβ express higher amounts of ECM molecules. **A.** Diagram demonstrating where isolated polycystic liver disease mutations are within cilia biogenesis. **B**. Immunohistochemistry of SMAD4 (cyan), pSMAD3$^{S423/S425}$ (magenta, arrows) and KRT19 (yellow) in isolated patient tissue with mutant *PRKCSH, SEC63,* or unknown. Scale bar=100µm. **C.** Immunoblots showing the expression of SMAD2/3 and the housekeeping protein GAPDH in H69 cells following 72 h of culture with either SIS3 or vehicle. **D.** Schematic demonstrating the experimental approach where H69 human cholangiocytes were stimulated with 10 ng/ml TGFβ for 16 h and differential gene expression of ECM genes and integrin transcripts following TGFβ stimulation (reads per kilobase of transcript per million reads mapped [RPKM] compared to vehicle control). **E.** mRNA expression of canonical TGFβ/SMAD target genes after stimulation with TGFβ.

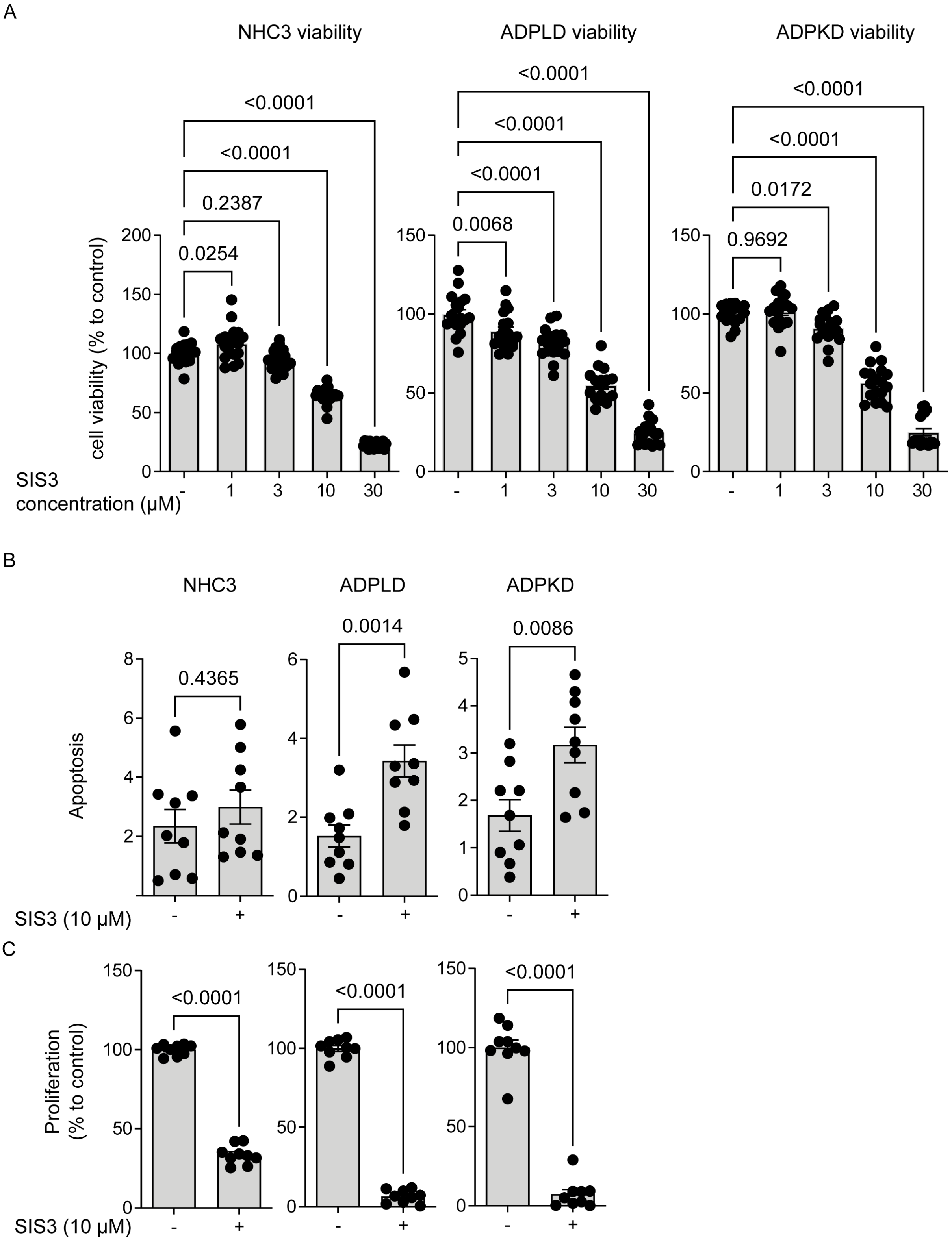

Figure S12: Human cholangiocytes are sensitive to treatment with SIS3. A. Normal human cholangiocytes (NHC3) or cells derived from liver cysts from patients with ADPLD or ADPKD treated with increasing concentrations of SIS3 (from 0-30 μM) and assayed for cell viability (n=16). **B.** Apoptosis and **C.** proliferation in normal human cholangiocytes (NHC3) or cells derived from liver cysts from patients with ADPLD or ADPKD when treated with 10μM SIS3 (n=9).

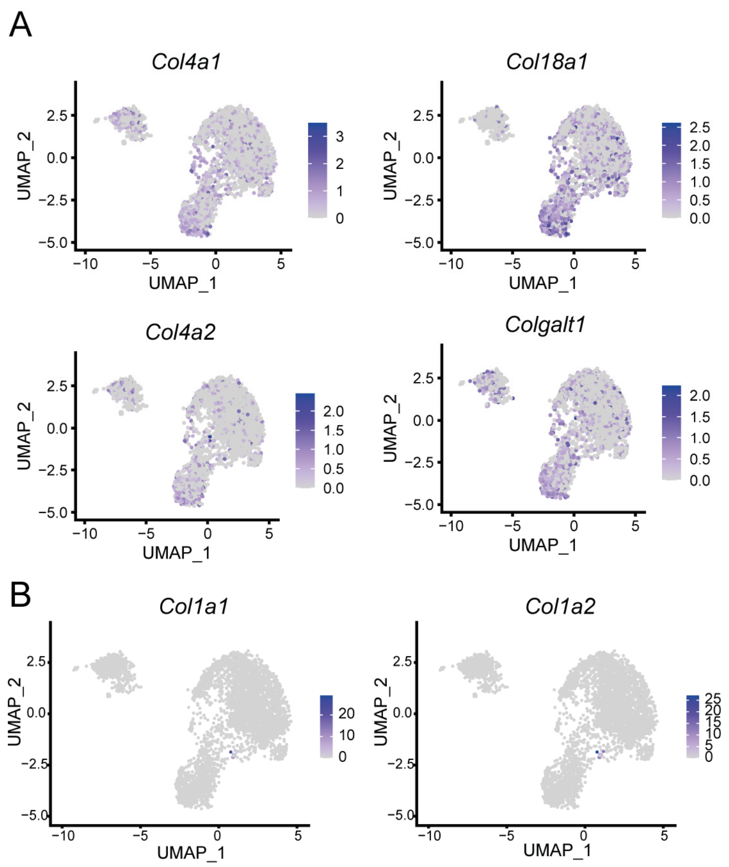

Figure S13: Mouse cystic BECs are enriched for extracellular matrix transcripts. A. UMAP showing expression of *Col4a1*, *Col4a2*, *Col18a1*, and *Colgalt1.* **B.** UMAP showing expression of *Col1a1 and Col1a2.*

A

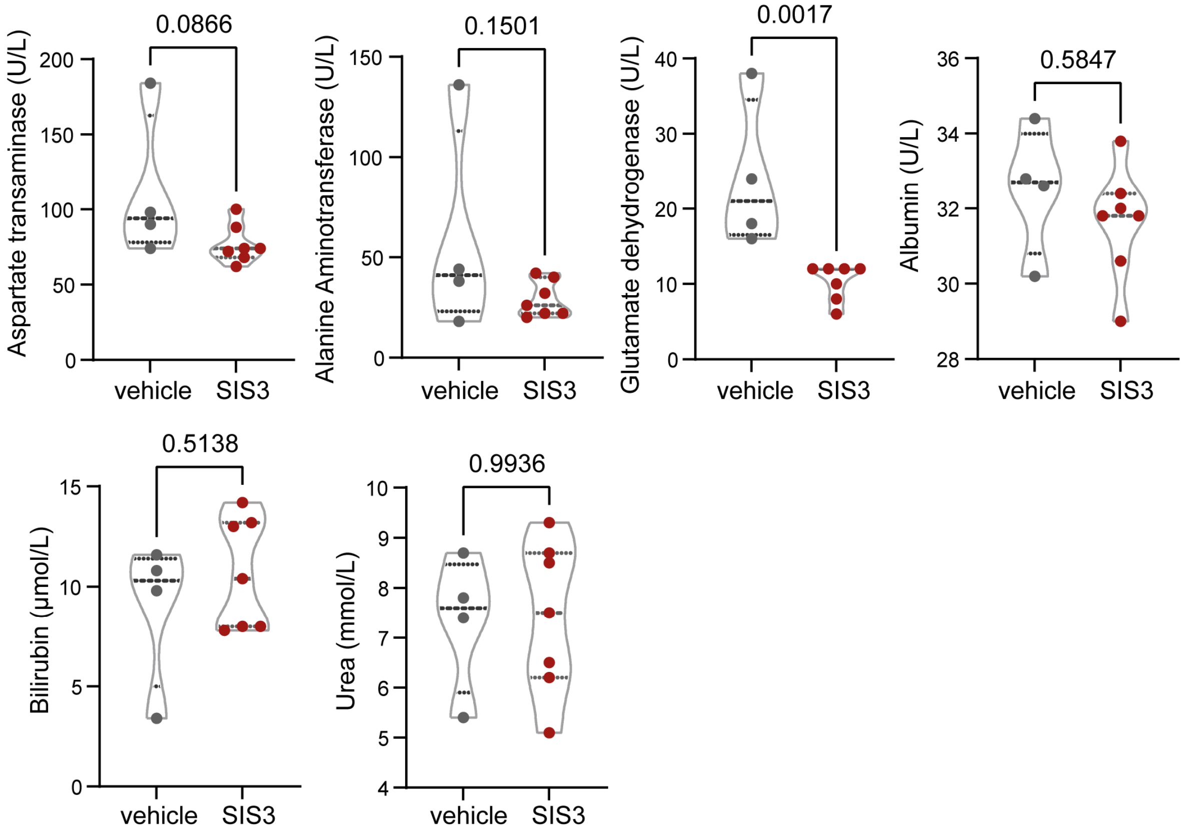

Figure S14: Blood biochemistry from mice treated with the SMAD3 inhibitor, SIS3. A. Blood serum biochemistry in *Wdr35*$^{-/-}$ cyst-bearing mice treated with the SMAD3-inhibitor, SIS3 (n=7) or vehicle (n=4) for 3 weeks.

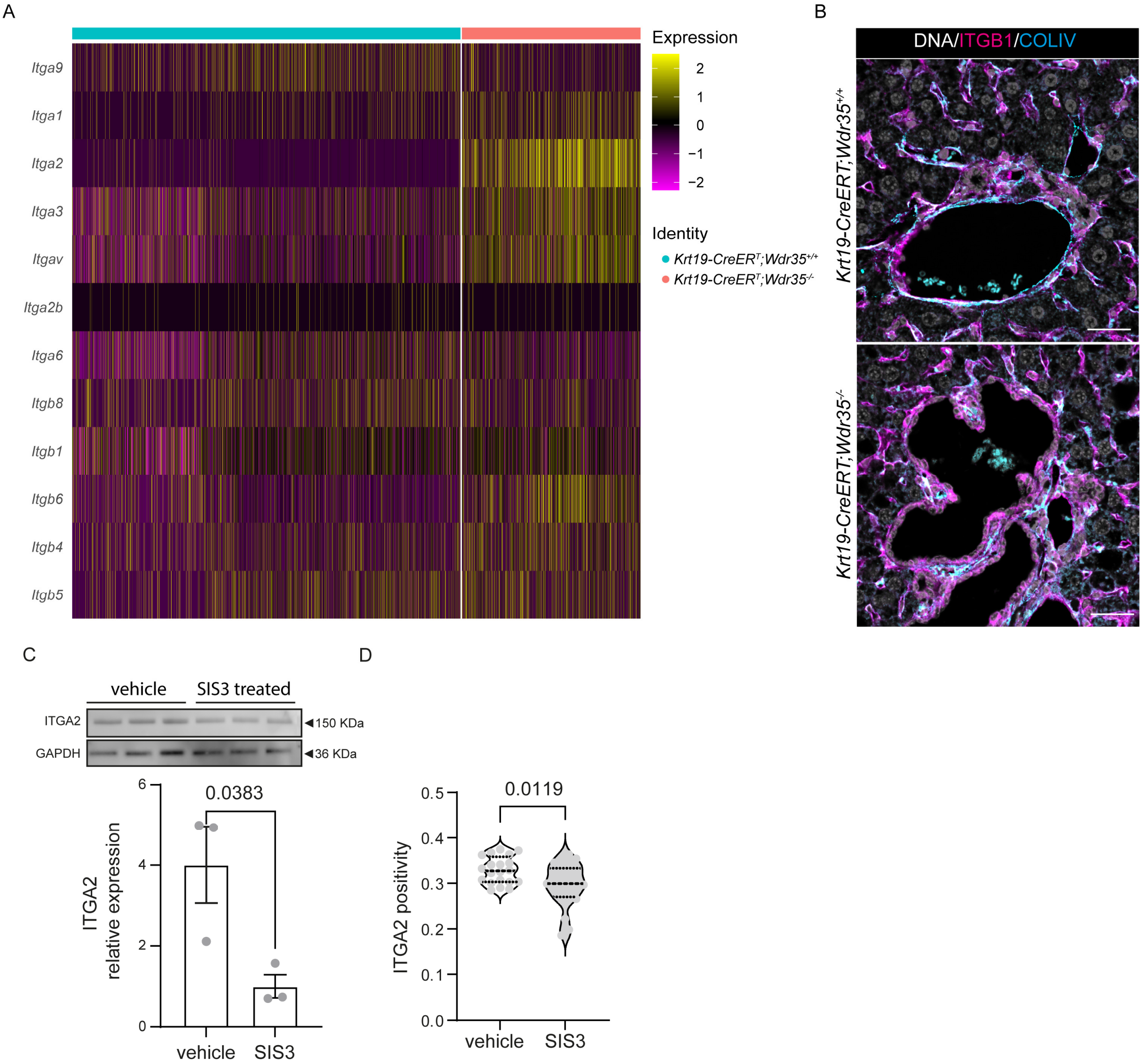

Figure S15: Cystic BECs express a profile of integrin molecules. A. Heatmap comparing the mRNA expression of all *Itga* and *Itgb* genes that are expressed in single-cell RNAseq analysis in BECs from *Wdr35*$^{+/+}$ and *Wdr35*$^{-/-}$ mice. **B.** Immunofluorescent staining of *Wdr35*$^{+/+}$ and *Wdr35*$^{-/-}$ for ITGB1 (magenta) and COLIV (cyan). DNA is in grey. Scale bar=100 µm. **C.** ITGA2 protein expression in 72-hour duct-to-cyst cultures after treatment with vehicle or SIS3. **D.** Quantification of ITGA2 immunohistochemistry in cyst-bearing Wdr35$^{-/-}$ animals treated with SIS3 or vehicle. n=100 biliary/cyst structures from five independent mice/treatment group.

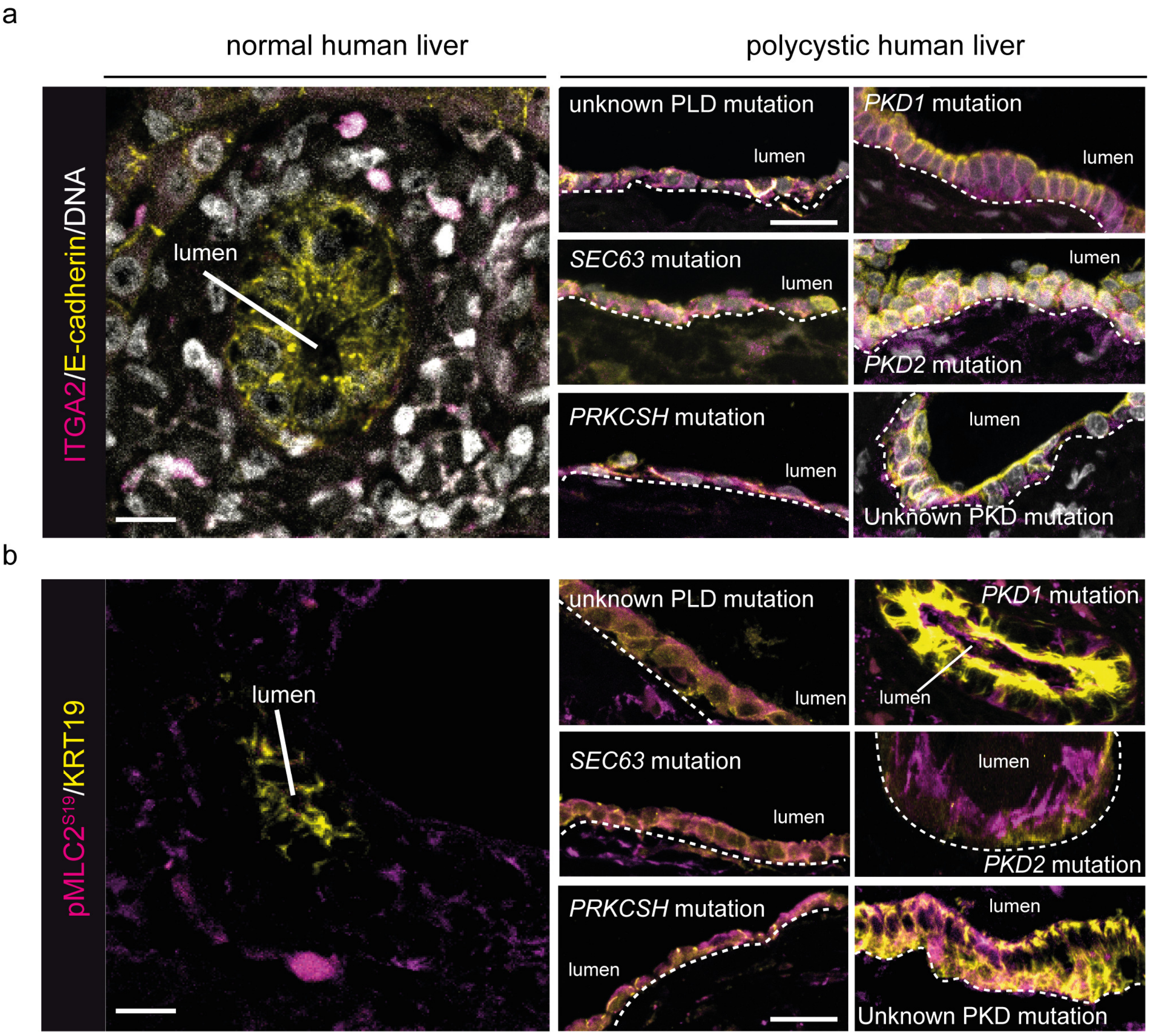

Figure S16: The formation of a pro-cystic state driven by integrin-α2β1 is common across genetic subtypes of PCLD. A. Immunofluorescent staining of human liver cysts with unknown PLD mutation, *SEC63* and *PRKCSH, PKD1, PKD2* or unknown PKD mutations for DNA (grey), ITGA2 (magenta) and E-cadherin (yellow) and **B.** pMLC2 (magenta) and E-cadherin (yellow), lower panels. Dotted line demarcates the boundary of the biliary epithelium. Scale bar for both normal and polycystic liver = 100 µm.

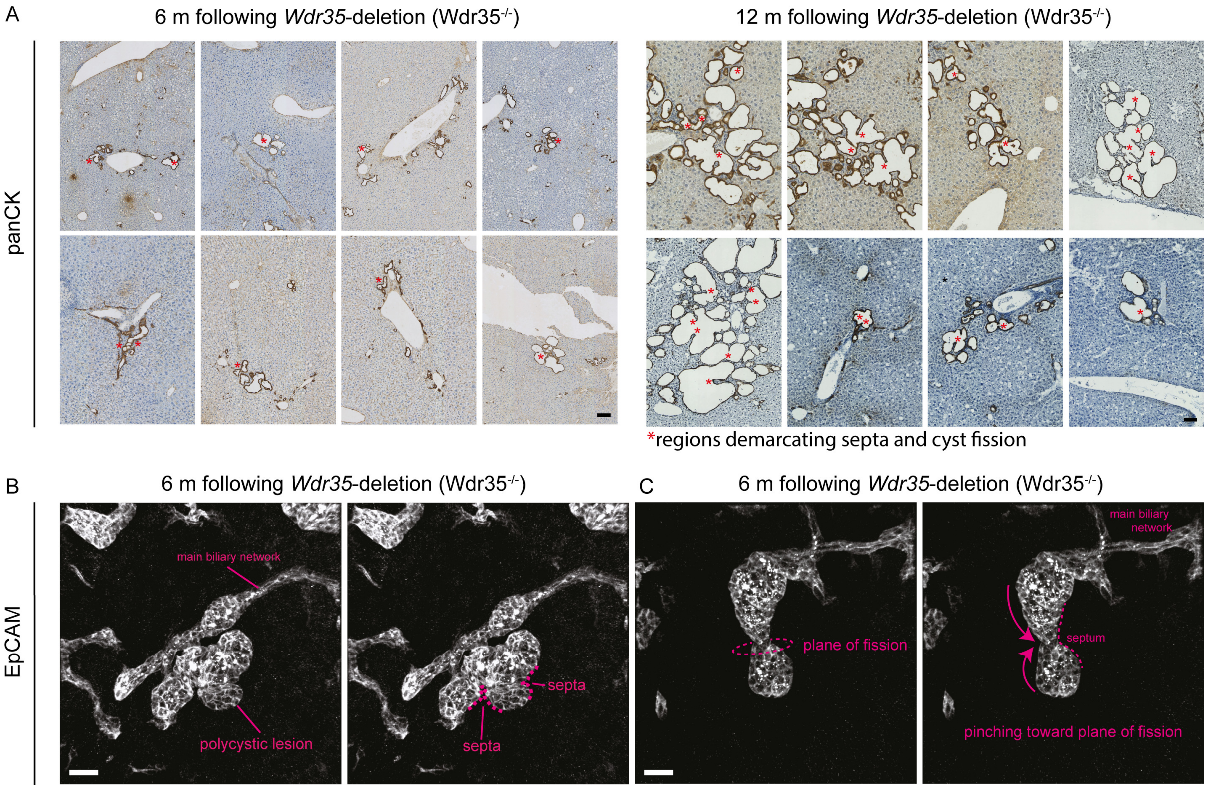

Figure S17: PCLD forms through the structural fission of pre-existing cysts. A. Immunohistochemical staining of *Wdr35*$^{-/-}$ liver 6 m or 12 m after tamoxifen treatment. Red asterisks denote regions in which cystic BECs form septa into the cyst lumen. **B, C.** Whole mount imaging of EpCAM-positive *Wdr35*$^{-/-}$ bile ducts demonstrating the formation of a cyst through cyst fission. Magenta lines denote the plane of fission. Scale bar = 100µm

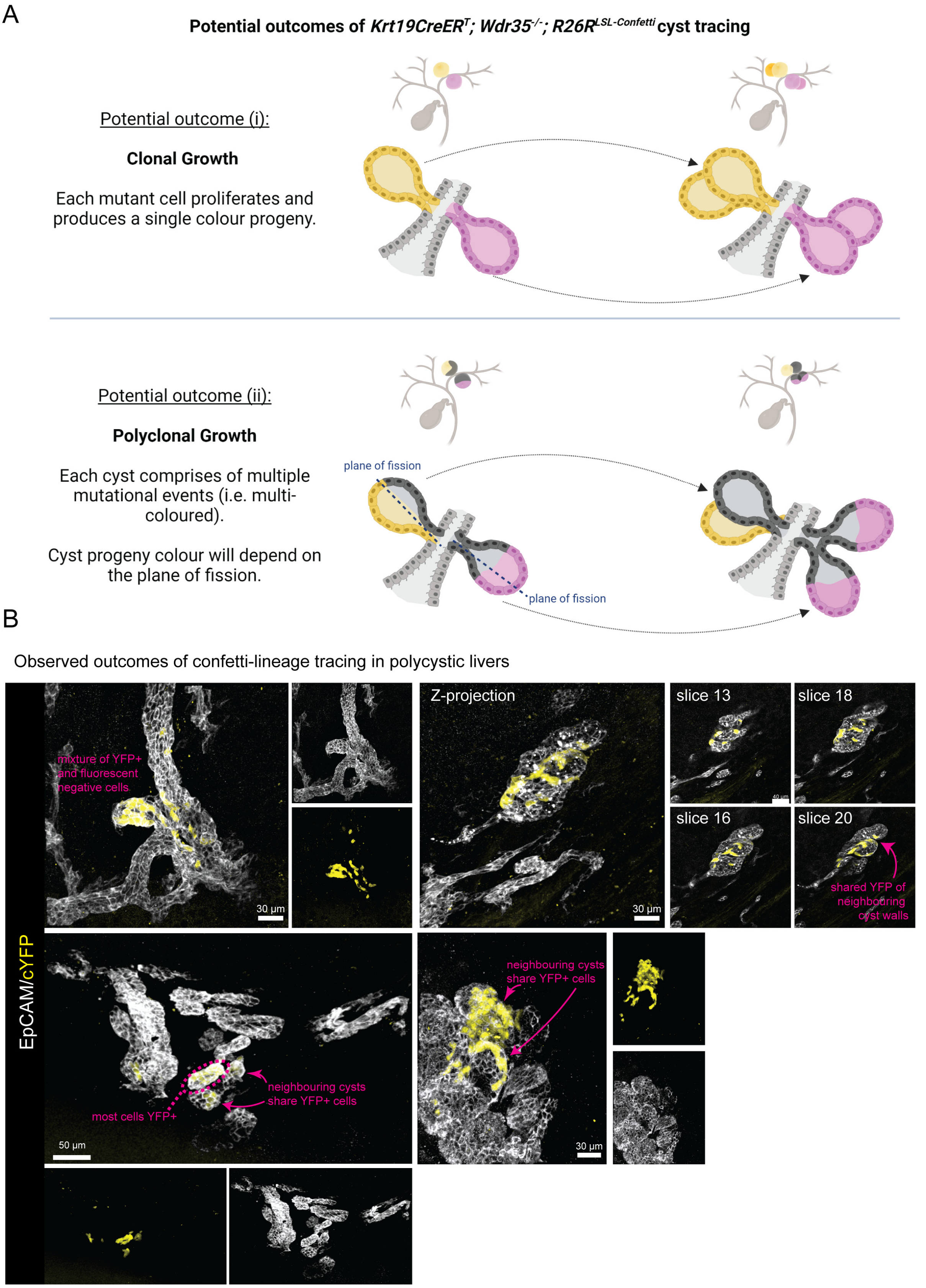

Figure S18: Examples of cystic fission using multi-colour lineage tracing. A. Schematic detailing the possible outcomes of multi-coloured lineage-tracing in *Krt19*^{CreERT};*Wdr35*$^{-/-}$;*R26R*$^{LSL-Confetti}$ cysts. **B.** FUnGI cleared whole mount confocal imaging of Confetti fluorophores (cYFP, yellow) in *Wdr35*$^{-/-}$ BECs, 6 months following tamoxifen administration demonstrating real-life examples of confetti-lineage tracing in polycystic livers.

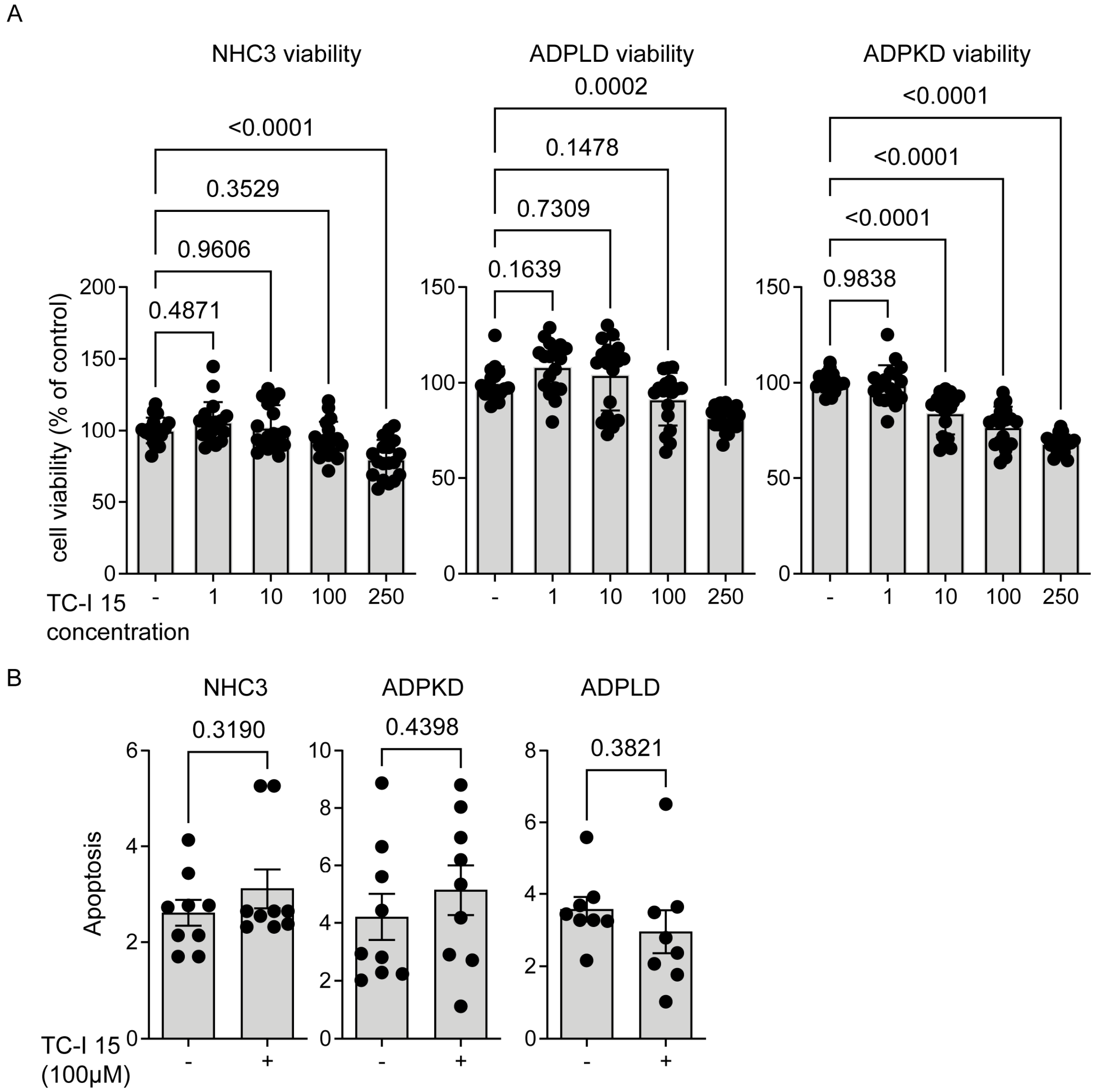

Figure S19: Human cells are sensitive to treatment with TC-I 15. A. Normal human cholangiocytes (NHC3) or cells derived from liver cysts from patients with ADPLD or ADPKD treated with increasing concentrations of TC-I 15 (from 0-250 μM) and assayed for cell viability (n=16). **B.** Apoptosis in normal human cholangiocytes (NHC3) or cells derived from liver cysts from patients with ADPLD or ADPKD when treated with 100μM TC-I 15 (n=9).

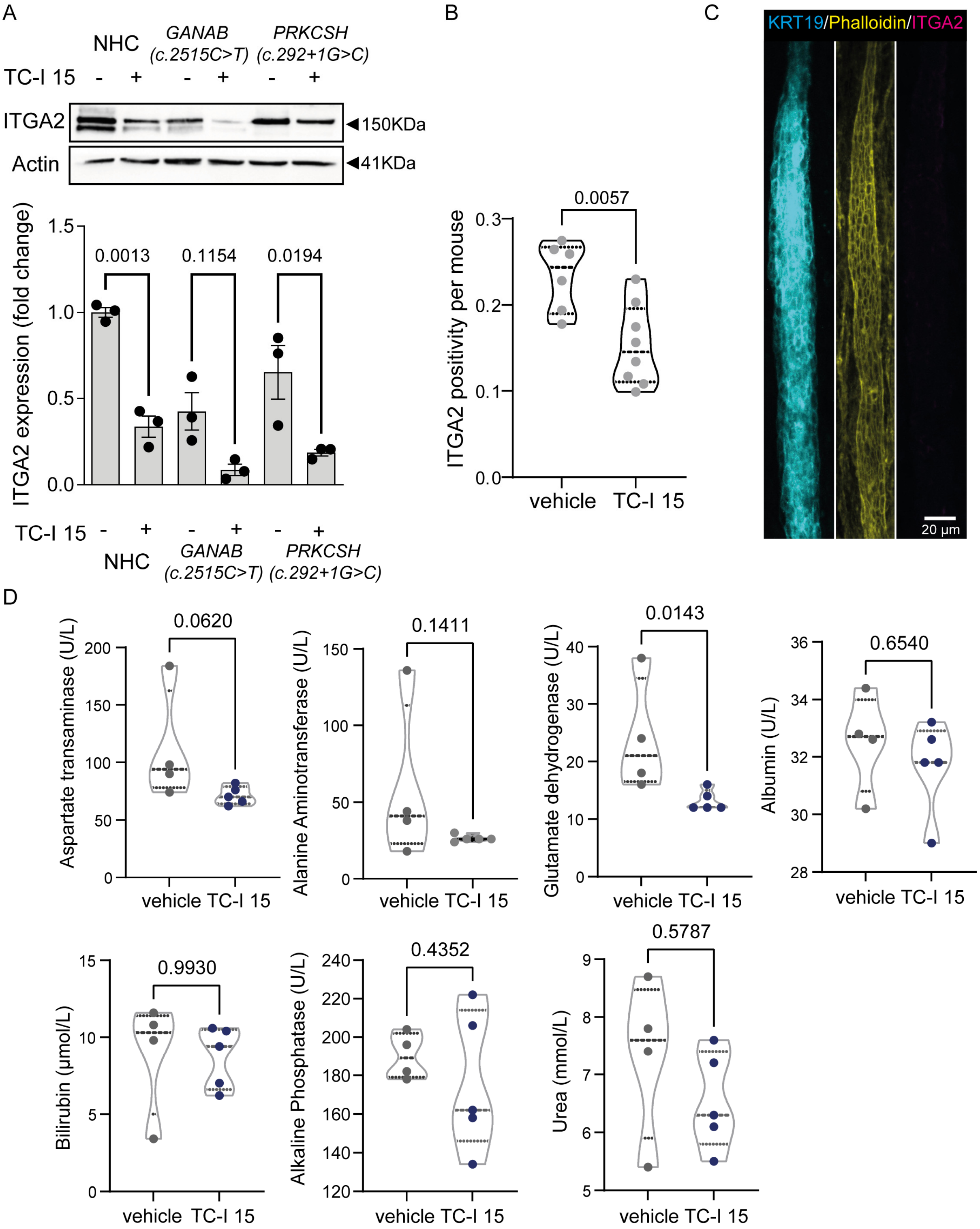

Figure S20: Integrin-α2β1 inhibition reduces ITGA2 abundance and alters blood biochemistry. A. Immunoblot of ITGA2 and actin in normal human cholangiocytes (NHC3) or cells derived from liver cysts from patients with either *GANAB* (*c. 2515C>T*) or *PRKCSH* (*c. 292+1G>C*) mutations and treated with TC-I 15 (n=3/group). **B.** Quantification of ITGA2 immunohistochemistry in cyst-bearing *Wdr35*$^{-/-}$ animals treated with vehicle (n=6) or TC-I 15 (n=8). **C.** Immunocytochemistry on freshly isolated bile ducts for KRT19 (cyan), F-actin (Phalloidin, yellow) and ITGA2 (magenta). **D.** Serum biochemistry from cyst bearing *Wdr35*$^{-/-}$ mice treated with vehicle alone (n=4 mice) or the integrin α2β1-inhibitor, TC-I 15 (n=5 mice).

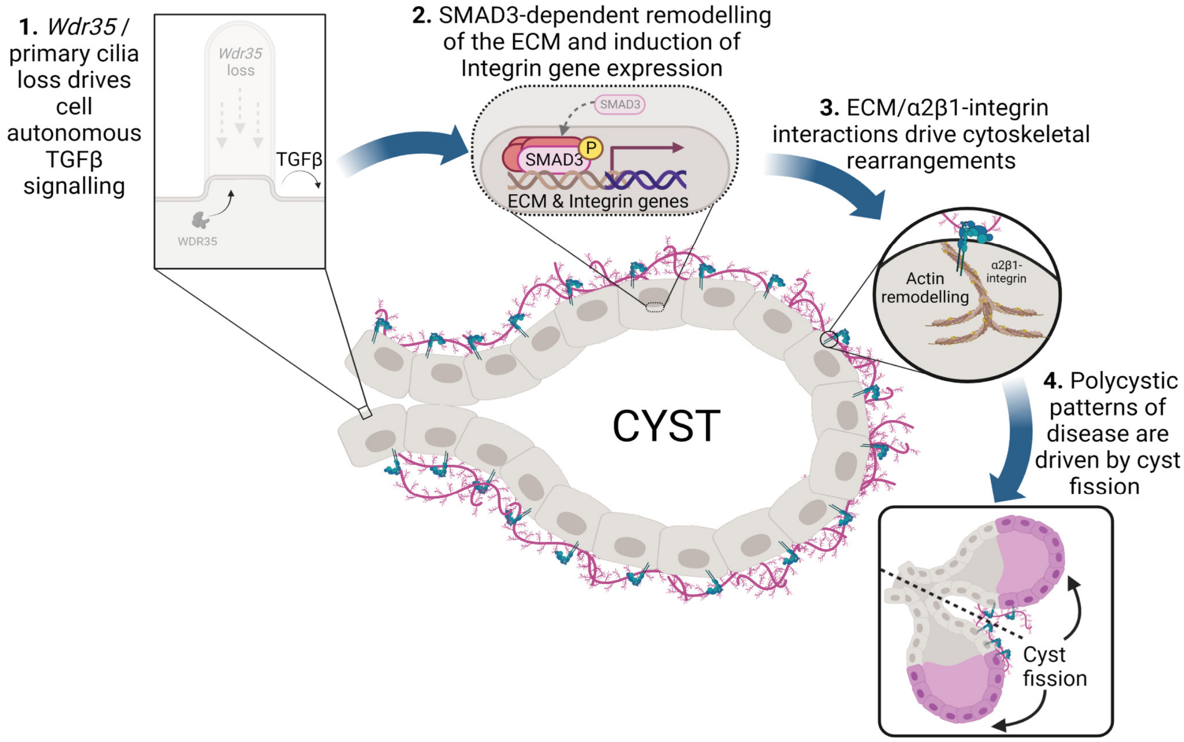

Figure S21: A schematic model demonstrating how liver cysts grow.

The following Excel files are available online:

Data file S1: Differentially expressed genes in wild-type BECs (cluster 1; c1) vs cystic BECs (cluster 2; c2) following single cell analysis.
Data file S2: Enriched GO and KEGG terms when comparing scRNA from wild-type BECs (wt) and cystic BECs (mut).
Data file S3: Differentially expressed genes in cystic BECs from SIS3- vs vehicle-treated cyst-bearing animals.
Data file S4: GOrilla and REViGO outputs from DEGs presented in data file S3.
Data file S5: Putative cell autonomous ligand-receptor interactions in wild-type BECs (cluster 1).
Data file S6: Putative cell autonomous ligand-receptor interactions in cystic BECs (cluster 2).
Data file S7: Clinical characteristics of human tissue samples.
Data file S8: Antibodies used in this study
Data file S9: Raw data contained within manuscript graphs.
